# Genetic deletion of fibroblast growth factor 14 recapitulates phenotypic alterations underlying cognitive impairment associated with schizophrenia

**DOI:** 10.1038/tp.2016.66

**Published:** 2016-05-10

**Authors:** T K Alshammari, M A Alshammari, M N Nenov, E Hoxha, M Cambiaghi, A Marcinno, T F James, P Singh, D Labate, J Li, H Y Meltzer, B Sacchetti, F Tempia, F Laezza

**Affiliations:** 1 Pharmacology and Toxicology Graduate Program, University of Texas Medical Branch, Galveston, TX, USA; 2 Department of Pharmacology and Toxicology, University of Texas Medical Branch, Galveston, TX, USA; 3 King Saud University Graduate Studies Abroad Program, King Saud University, Riyadh, Saudi Arabia; 4 Neuroscience Institute Cavalieri Ottolenghi, Turin, Italy; 5 Department of Neuroscience, University of Torino, Turin, Italy; 6 Department of Mathematics, University of Houston, Houston, TX, USA; 7 Department of Psychiatry and Behavioral Sciences, Northwestern University Feinberg School of Medicine, Chicago, IL, USA; 8 Mitchell Center for Neurodegenerative Diseases, The University of Texas Medical Branch, Galveston, TX, USA; 9 Center for Addiction Research, The University of Texas Medical Branch, Galveston, TX, USA

## Abstract

Cognitive processing is highly dependent on the functional integrity of gamma-amino-butyric acid (GABA) interneurons in the brain. These cells regulate excitability and synaptic plasticity of principal neurons balancing the excitatory/inhibitory tone of cortical networks. Reduced function of parvalbumin (PV) interneurons and disruption of GABAergic synapses in the cortical circuitry result in desynchronized network activity associated with cognitive impairment across many psychiatric disorders, including schizophrenia. However, the mechanisms underlying these complex phenotypes are still poorly understood. Here we show that in animal models, genetic deletion of fibroblast growth factor 14 (*Fgf14*), a regulator of neuronal excitability and synaptic transmission, leads to loss of PV interneurons in the CA1 hippocampal region, a critical area for cognitive function. Strikingly, this cellular phenotype associates with decreased expression of glutamic acid decarboxylase 67 (GAD67) and vesicular GABA transporter (VGAT) and also coincides with disrupted CA1 inhibitory circuitry, reduced *in vivo* gamma frequency oscillations and impaired working memory. Bioinformatics analysis of schizophrenia transcriptomics revealed functional co-clustering of *FGF14* and genes enriched within the GABAergic pathway along with correlatively decreased expression of *FGF14*, *PVALB*, *GAD67* and *VGAT* in the disease context. These results indicate that *Fgf14*^−/−^ mice recapitulate salient molecular, cellular, functional and behavioral features associated with human cognitive impairment, and *FGF14* loss of function might be associated with the biology of complex brain disorders such as schizophrenia.

## Introduction

Psychiatric diseases such as schizophrenia, depression and bipolar disorder are associated with cognitive deficits thought to arise from an imbalance between the excitatory and inhibitory (E/I) tone in cortical circuits.^1, 2^ Fast-spiking GABAergic parvalbumin (PV) interneurons have a fundamental role in maintaining E/I balance, controlling excitability and shaping the synaptic plasticity of principal neurons.^3^ Even though disrupted function of other types of inhibitory neurons has been associated with the etiology of brain disorders, changes in PV neurons are one of the most consistent associations. In animal models, reduced function of PV neurons results in desynchronized network activity, decreased gamma frequency oscillations and cognitive deficits, phenotypes that mimic clinical presentation and post-mortem tissue studies of virtually all human brain disorders associated with disrupted cognition.^1, 4, 5, 6, 7, 8, 9, 10^

In schizophrenia and bipolar patients, the number of PV interneurons and the expression level of molecular components of GABAergic synapses, such as glutamic acid decarboxylase 67 (GAD67) and vesicular gamma-amino-butyric acid (GABA) transporter (VGAT), are found decreased in post-mortem brains leading to the ‘GABA hypofunction hypothesis' as a potential etiology.^1, 11, 12, 13, 14, 15, 16^ Yet, the molecular understanding of how such a detrimental loss of the GABAergic system might lead to corrupted cortical networks manifesting in disease, remains poorly explored.

Recent large-scale genome-wide association studies identified *FGF14* as locus of single-nucleotide polymorphisms and as such a potential disease-associated gene for schizophrenia, bipolar disease, depression, epilepsy and addictive behaviors,^17, 18, 19, 20, 21, 22, 23, 24, 25, 26, 27^ corroborating initial reports of missense mutations in *FGF14* as genetic links to the neurodegenerative disorder, spinocerebellar ataxia 27.^28, 29^ These results highlight the possibility of an as yet undiscovered and pivotal role for the *FGF14* gene in psychiatric disorders.

Highly expressed in the central nervous system, FGF14 is an accessory protein of voltage-gated Na+ (Nav) channels at the axonal initial segment (AIS),^30, 31, 32, 33, 34, 35, 36^ a regulator of neuronal excitability,^30, 37, 38, 39^ a presynaptic organizer of glutamatergic synapses,^40, 41^ a scaffolding molecule for kinase signaling pathways^33, 34, 42, 43^ and a regulator of synaptic plasticity.^41, 44, 45^ At the circuitry and behavioral level, genetic deletion of FGF14 in *Fgf14*^−/−^ mice results in presynaptic structural deficits of CA3–CA1 hippocampal synapses, decreased long-term potentiation, and cognitive deficits, aberrant responses to epileptic agents and decreased threshold for seizure induction.^41, 44, 45^ Furthermore, *Fgf14*^−/−^ mice exhibit abnormal locomotor activity explained by a reduced response to dopamine receptor D2 agonists in the basal ganglia.^45^ Both phenotypes in the hippocampus and the basal ganglia could imply an impaired GABAergic tone supporting the emerging view of a tight correlation between the dopamine and the GABAergic systems.^46, 47^ Yet, evidence of FGF14 control of GABAergic inhibitory transmission is lacking.

Using a combination of confocal microscopy, patch-clamp electrophysiology, *in vivo* local field potential recordings and behavioral studies, we discovered that genetic deletion of *Fgf14* in rodents leads to a reduced number of PV interneurons, decreased expression of GAD67 and VGAT, and reduced GABAergic transmission in CA1 pyramidal neurons associated with impaired gamma oscillations and working memory. Further, bioinformatics analysis of schizophrenia transcriptomics confirms FGF14 functional clustering with GABAergic synaptic signaling and identifies genetic covariance of *FGF14, PVALB*, *GAD67* and *VGAT* in the disease, supporting *Fgf14*^−/−^ mice as an attractive model to interrogate the biology of complex brain disorders associated with disrupted cognitive circuitry such as schizophrenia.

## Materials and Methods

### Animals

The primary *Fgf14*^*−/−*^ colony was maintained at the animal facilities of the University of Texas Medical Branch and of the University of Torino following approved protocols. Genotypes were confirmed by in-house PCR analysis or by Charles River Laboratories International (Houston, TX, USA). Description of the animal husbandry is given in the Supplementary Material.

### Immunofluorescence

Brain tissue derived from *Fgf14*^−/−^ and *Fgf14*^+/+^ 4- to 5-month-old male littermates was processed for immunofluorescence staining using either 4% paraformaldehyde or liquid nitrogen vapor fixation followed by permeabilization, blocking, and primary and Alexa-conjugated secondary antibody staining as previously described^48^ and outlined in the Supplementary Material.

### Data acquisition and image analysis

Multichannel confocal images were acquired using a Zeiss LSM-510 META (Carl Zeiss Microscopy, Jena, Germany) confocal microscope as previously described.^49^ Detailed information on image quantification can be found in the Supplementary Material.

### Western blotting

Tissue homogenate preparation and western blotting were carried out as previously described^50^ and outlined in the Supplementary Material.

### Whole-cell patch-clamp electrophysiology

Horizontal hippocampal slices were prepared from 4- to 5-month-old *Fgf14*^−/−^ and *Fgf14*^+/+^ male littermates, and whole-cell patch-clamp recordings were performed at room temperature from CA1 pyramidal neurons using standard parameters previously described^51, 52^ and outlined in the Supplementary Material.

### *In vivo* electroencephalogram

Extracellular field potentials were recorded in freely behaving mice using stainless steel electrodes stereotaxically implanted in the CA1 of the right hippocampus.^53^ Spectrograms were calculated using the software NeuroExplorer (Nex Technologies, Madison, AL, USA).

### Behavioral tests

Tests were performed in *Fgf14*^−/−^ and *Fgf14*^+/+^ 2- to 4-month-old males using the 4–8 version of the eight-arm radial maze.^54^ A detailed description of behavioral experiments and analysis are part of the Supplementary Material.

### Human studies

As a computational gene co-expression search engine, SEEK (http://seek.princeton.edu/) was applied to identify a gene network co-expressed with FGF14. Functional annotation and pathway enrichment was performed with a built-in Gene Ontology and KEGG terms. Enriched data sets were downloaded from NCBI Gene Expression Omnibus, and the differential gene expression was analyzed by R Limma packages described in the Supplementary Material. We also applied GeneMANIA and STRING to the analysis of pathway enrichment and protein–protein interaction for the co-expressed genes of FGF14 in hippocampus. The method and result are detailed in the Supplementary Material.

## Results

**Figure 1:**
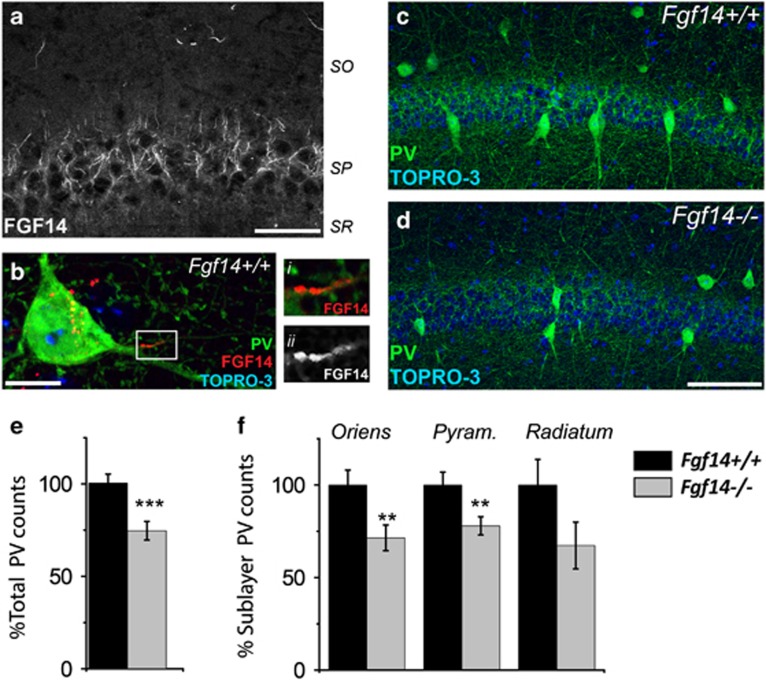
Genetic deletion of *Fgf14* results in structural changes in the CA1 parvalbumin (PV) interneurons. (**a**) FGF14 immunoreactivity is detectable at the axonal initial segment (AIS) of cells in CA1. (**b**) FGF14 (red) expressed in the soma and AIS of PV interneurons (green), i and ii represent zooms of the boxed area. (**c**, **d**) PV interneurons in the CA1 region of *Fgf14*^*+/+*^ and *Fgf14*^−/−^ mice and respective higher resolution views of PV somas (i and ii). (**e**, **f**) Quantification of total PV interneurons in CA1 (380 cells in *Fgf14*^*+/+*^ and 282 in *Fgf14*^−/−^), and in specific subfields (oriens, pyramidalis and radiatum). Data represent mean±s.e.m., ***P<0.001; **P<0.02; *P<0.05 statistical differences were assessed by Student's *t*-test or non-parametric Mann–Whitney test. Scale bars, 40 μm (**a**); 10 μm (**b**); 100 μm (**d**).

Anatomical abnormalities found in the CA1 hippocampal region of post-mortem brains from schizophrenia patients have been identified as an index of the disease severity and treatment responsiveness.^55^ These studies along with the reported association between hippocampal PV neurons with cognitive function in the normal brain and in schizophrenia, prompted us to begin our investigations in the CA1 hippocampal region.^15, 16, 46, 47, 55^ Thus, we first asked whether FGF14 was expressed in PV interneurons in the CA1 region. Confocal imaging confirmed that FGF14 is expressed at the AIS of cells across all CA1 sublayers (Figure 1a), including PV-positive neurons (Figure 1b). Therefore, we posited whether genetic ablation of *Fgf14* might have physiological consequences for these inhibitory neurons.

To test this hypothesis, we first examined whether the overall number of PV interneurons in the CA1 area was affected by genetic loss of FGF14. Cell count quantification based on immunofluorescence staining revealed that the total number of PV-positive cells was significantly reduced in *Fgf14*^−/−^ mice compared with wild-type controls (Figures 1c–e; 74.21%±5.02, 100%±4.89, *P*<0.001) with a most pronounced and significant reduction in the stratum oriens (SO; 71.42%±6.96, 100%±8.10, *P*<0.05) and in stratum pyramidalis (SP; 77.94%±4.96, 100%±7.06, *P*<0.05; Figure 1f). Notably, the expression level of PV per cell was not significantly different across groups (*P*=0.78, *n*=4 littermate mice; Supplementary Figures 1F–I); the neuropil composed of PV-positive neurites in either SP or SO was also unaffected by *Fgf14* deletion (*P*=0.49 and 0.28; Supplementary Figures 1A–E). Furthermore, the total number of cells in the CA1 SP, which primarily includes pyramidal neurons, was also unchanged (*P*=0.24; Figures 1j–l). Thus, we concluded that deletion of *Fgf14* leads to a cell- and subfield-specific reduction in the number of PV neurons in the CA1 hippocampal region.

**Figure 2:**
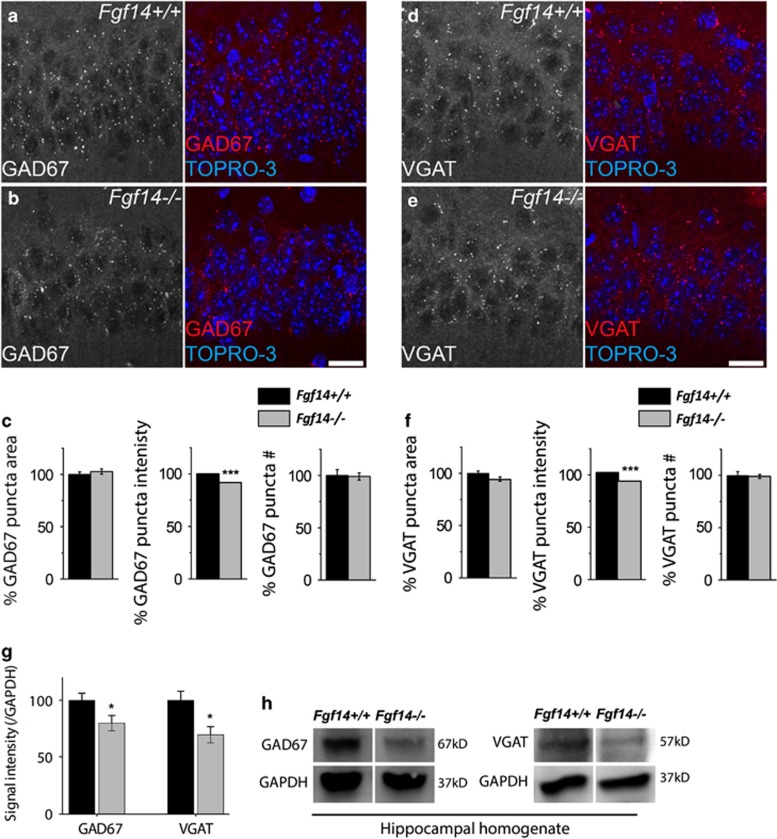
The effect of *Fgf14* genetic ablation on GABAergic presynaptic proteins in the hippocampal CA1 region. (**a**, **b**) GAD67 representative puncta in CA1 stratum pyramidalis (SP) at high magnification. (**c**) Quantification of GAD67 puncta area, puncta intensity and puncta number in the indicated genotypes. (**d**, **e**) Vesicular GABA transporter (VGAT) representative puncta in CA1 SP at high magnification. (**f**) Quantification of VGAT puncta area, puncta intensity and puncta number in the indicated genotypes. (**g**) Quantitative western blot analysis of GAD67 and VGAT from the hippocampus. (**h**) Immunoblot detection of GAD67 and VGAT in whole hippocampal homogenates from *Fgf14*^−/−^mice and *Fgf14*^*+/+*^ controls. Data represent mean±s.e.m., ***P<0.001; **P<0.02; *P<0.05 statistical differences were assessed by Student's *t*-test or non-parametric Mann–Whitney test. Scale bars, 20 μm (**a**, **d**). GABA, gamma-amino-butyric acid; GAD67, glutamic acid decarboxylase 67.

PV interneurons are the primary source of GABAergic synapses in the CA1 region. Reduction in GABA synthesis and its synaptic release machinery accompany loss of PV neurons in schizophrenia and other psychiatric disorders.^4, 5, 6^ Consistent with this, GAD67 mean fluorescence intensity in PV soma, the primary source of the enzyme pool, and in puncta across SP was significantly reduced in *Fgf14*^−/−^ mice compared with littermate control (89.85%±3.08, 100%±3.96, *P*<0.05; and 91.86%±0.10, 100%±0.08, *P*<0.0001, respectively; Supplementary Figures 2A–D and Figures 2a–c). The area per puncta and total puncta number, though, were unchanged (*P*=0.10 and 0.88, respectively; Figure 2c). An additional marker and key regulatory protein at presynaptic GABAergic inputs is VGAT, the vesicular transporter that loads presynaptic vesicles with GABA, another marker associated with brain disorders.^56, 57, 58^ Along with GAD67, the mean fluorescence intensity of VGAT in PV soma and its mean content per puncta in SP were significantly decreased in *Fgf14*^−/−^ mice compared with littermate controls (88.12%±3.06 vs 100%±3.18, *P*<0.01, *n*=4 littermate mice and 91.92%±0.14 vs 100%±0.135, *P*<0.001, *n*=3 littermate mice, respectively; Supplementary Figures 2E–H and Figures 2d–f), while puncta area and number were unchanged (*P*=0.66 and 0.79, *n*=3 mice; Figure 2f). Consistent with these results, Western blot analyses of hippocampal cell lysate revealed a significant reduction of GAD67 and VGAT protein content in *Fgf14*^−/−^ animals compared with wild type (Figures 2g and h).

**Figure 3:**
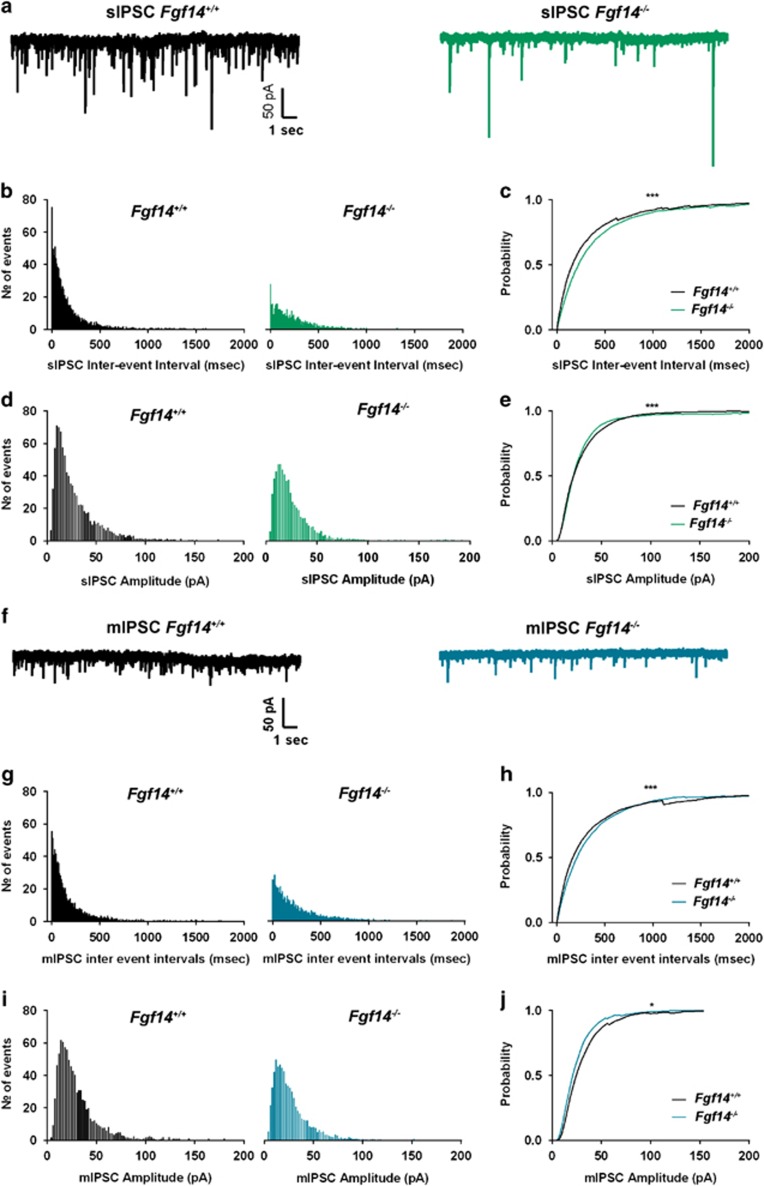
Genetic deletion of *Fgf14* impairs GABAergic transmission in the CA1 region. Representative traces of whole-cell patch-clamp recordings showing effect of *Fgf14* ablation on spontaneous inhibitory postsynaptic current (sIPSCs) (**a**) and miniature inhibitory postsynaptic current (mIPSCs) (**f**). (**b**) Inter-event-interval distribution of spontaneous GABAergic events in *Fgf14*^*+/+*^ (*n*=8 cells) and *Fgf14*^−/−^(*n*=10 cells) mice. (**c**) Inter-event-interval cumulative distribution plot for *Fgf14*^*+/+*^ and *Fgf14*^−/−^(sIPSCs; ***P<0.001, Kolmogorov–Smirnov test). (**d**) Amplitude distribution of spontaneous GABAergic events in *Fgf14*^*+/+*^ (*n*=8 cells) and *Fgf14*^−/−^ (*n*=10 cells) mice. (**e**) Amplitude cumulative distribution plot for *Fgf14*^*+/+*^ and *Fgf14*^−/−^ sIPSCs (***P<0.001; Kolmogorov–Smirnov test). (**g**) Inter-event-interval distribution of miniature GABAergic events in *Fgf14*^*+/+*^ (*n*=6 cells) and *Fgf14*^−/−^ (*n*=7 cells) mice. (**h**) Inter-event-interval cumulative distribution plot for *Fgf14*^*+/+*^ and *Fgf14*^−/−^ mIPSCs (***P<0.001 with Kolmogorov–Smirnov test). (**i**) Amplitude distribution of miniature GABAergic events in *Fgf14*^*+/+*^ (*n*=6 cells) and *Fgf14*^−/−^ (*n*=7 cells) mice. (**j**) Amplitude cumulative distribution plot for *Fgf14*^*+/+*^ and *Fgf14*^−/−^ sIPSCs (*P<0.05; Kolmogorov–Smirnov test).

PV interneurons contribute greatly to the integrity of the GABAergic circuit in the CA1 region, posing the question of whether *Fgf14* deletion might result in functional consequences for inhibitory transmission. Using patch-clamp electrophysiology, we recorded spontaneous and miniature inhibitory postsynaptic currents (sIPSCs and mIPSCs, respectively) from visually identified CA1 pyramidal neurons in *Fgf14*^−/−^ and wild-type control mice (Figures 3a and f). We found that genetic deletion of *Fgf14* led to a rightward shift in the probability distribution of sIPSC frequency (apparent loss in the higher frequency domain) accompanied by a reduction in the largest- and smallest-size synaptic event population (Figures 3c–e; *P*<0.001, Kolmogorov–Smirnov test). Spontaneous IPSCs are action potential-driven synaptic events and as such represent a compound readout of the firing status of presynaptic interneurons and their neurotransmitter release machinery. To examine GABA release mechanisms independently from interneuron spontaneous firing, we recorded mIPSCs in the presence of tetrodotoxin and found that, similarly to sIPSCs, the frequency distribution histograms showed lowered probability of short inter-event intervals in *Fgf14*^−/−^ when compared with wild-type control mice (Figures 3f–h, *P*<0.001 with Kolmogorov–Smirnov test). This phenotype was paralleled by a loss of large- and small-amplitude mIPSCs in *Fgf14*^−/−^ compared with wild-type control mice (Figures 3f, i and j). In both sIPSCs and mIPSCs, the averaged sIPSC frequency and amplitude between the two groups were not significantly different (Supplementary Figure 3) and no changes in rise and decay time were found across synaptic events (both sIPSCs and mIPSCs) of different genetic groups (Supplementary Figure 4). Thus, genetic deletion of *Fgf14* leads to functional changes in the CA1 inhibitory circuitry that support the structural alterations demonstrated in Figures 1 and 2. Moreover, it suggests loss of interneuron firing (shift in sIPSC frequency) and pre- and postsynaptic modifications at GABA synapses (shift in frequency and amplitude distribution of mIPSCs, respectively).

**Figure 4:**
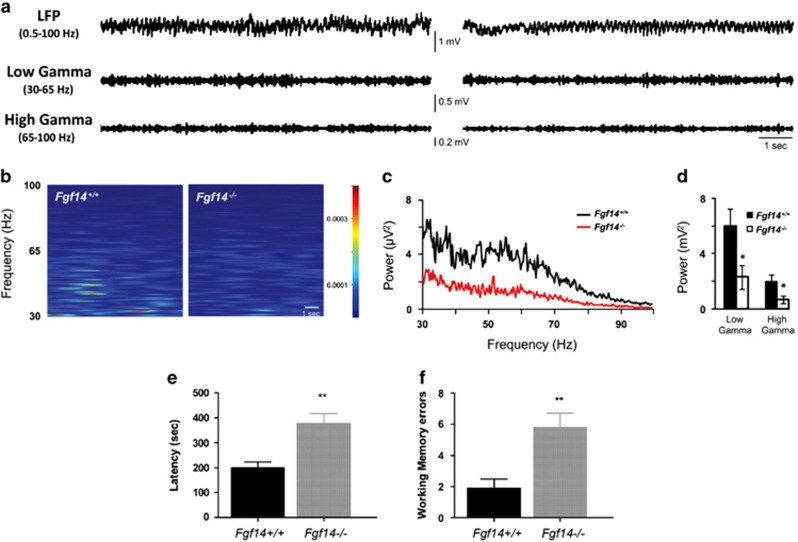
Genetic deletion of *Fgf14* reduces gamma frequency and affects working memory. (**a**) Representative traces of 10-s *in vivo* electroencephalogram recordings in the CA1 region of hippocampus (local field potential; LFP) in *Fgf14*^*+/+*^ (left) and *Fgf14*^−/−^ mice (right); filtered traces within low- and high-gamma band are also shown. (**b**) Spectrogram analysis of the above-mentioned traces in the gamma range (30–100 Hz). (**c**) Mean power spectral density of CA1 activity showing a marked decrease in gamma power in *Fgf14*^−/−^ (*n*=7) with respect to *Fgf14*^*+/+*^ (*n*=7) mice, as revealed by power analysis within both low- and high gamma (**d**). (**e**) *Fgf14*^−/−^ mice required a longer time to perform the eight-arm maze test (*n*=20 wild type and *n*=19 *Fgf14*^−/−^; *P*<0.001, *t*-test). (**f**) Analysis of working memory errors committed during the test day showed a significant difference between genotypes (*P*<0.05, *t*-test). Data are expressed as mean±s.e.m. *P<0.05.

Reduced PV neuron function can desynchronize the CA1 network resulting in reduced gamma oscillations and impaired cognition. Thus, we postulated that gamma oscillations might be impaired upon ablation of *Fgf14*. To test this, gamma oscillations were recorded in the CA1 SR layer in *Fgf14*^−/−^ and control mice by *in vivo* local field potential (Figure 4a). Spectral analysis within the 30–100 Hz range revealed that *Fgf14*^−/−^ mice had a strong reduction in gamma oscillation power (2.94±0.11 μV^2^, *n*=7 compared with 7.92±0.17 μV^2^, *n*=7 in wild-type mice, *P*<0.05; Figures 4b and c) with both slow (low gamma, 30–65 Hz) and fast (high gamma, 65–100 Hz) gamma oscillations significantly impaired in *Fgf14*^−/−^ mice compared with wild type (*Fgf14*^−/−−/−^, 2.29±0.85 and 0.65 ±0.25 μV^2^; wild-type control, 5.97±1.2 and 1.95±0.5 μV^2^; Figure 4d). Thus, consistent with disrupted GABAergic transmission, spectral analysis confirmed that FGF14 is required for the integrity of the cognitive circuitry. The combination of phenotypes observed in *Fgf14*^−/−^ mice (Figures 1) has been associated with deficits in spatial working memory in animal models and patients afflicted with schizophrenia.^1, 58, 59^ Thus, we next evaluated the spatial working memory performance of *Fgf14*^−/−^ mice using the eight-arm maze test. Analysis of the latency to perform the task showed that *Fgf14*^−/−^ mice required a longer time to complete the task than wild type (*n*=20 wild type and *n*=19 *Fgf14*^−/−^, *P*<0.001; Figure 4e). Furthermore, revisiting errors (Figure 4f), which are directly linked to working memory performance, were more frequent in *Fgf14*^−/−^ mice than in wild types (*P*<0.05), corroborating the cellular and functional phenotypes associated with genetic deletion of *Fgf14*.

**Figure 5:**
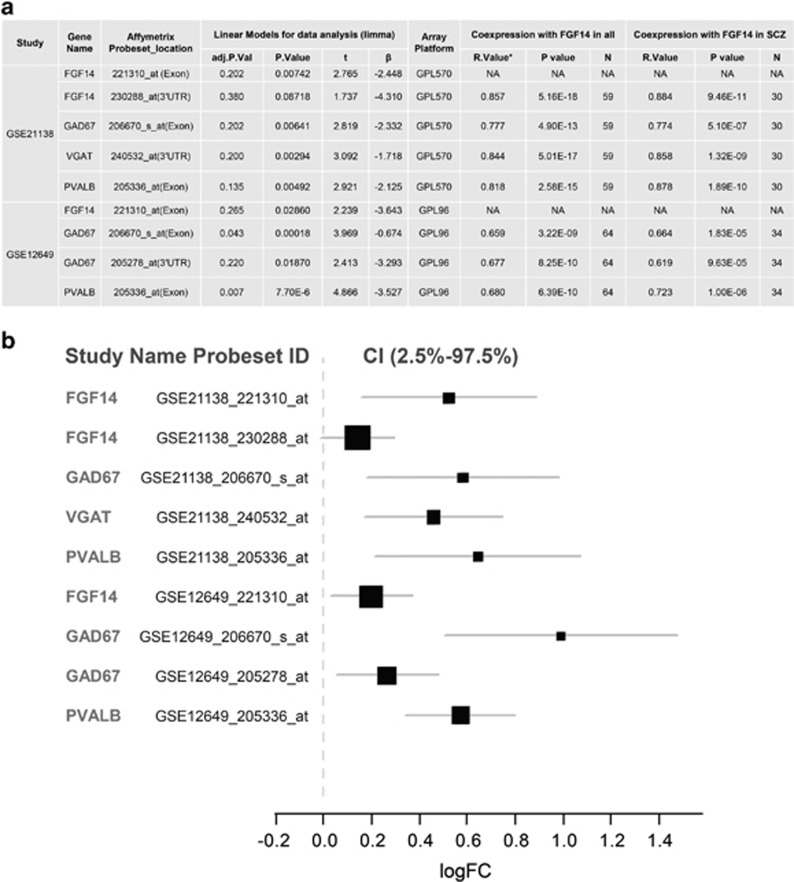
Differential gene expression and correlation of *FGF14*, *PVALB*, *VGAT* and *GAD67* in post-mortem control and schizophrenia samples. (**a**) The GSE21138 and GSE12649 data sets are both derived from previous studies^60, 61^ and deposited in NCBI Gene Expression Omnibus (GEO). Owing to significant deviation from the mean (>2 s.d.) in *FGF14* gene expression (221310_at), five samples were removed from the GSE12649 data set. GPL96 represents Affymetrix Human Genome U133A Array; GPL570 represents Affymetrix Human Genome U133 Plus 2.0 Array. GPL96 had no probeset selected for VGAT (SLC32A1). The original *P*-value was adjusted by Benjamini and Hochberg (false discovery rate). The *R*-value represents a Pearson Correlation with significance at the 0.01 level (two-tailed). (**b**) Forest plot illustrates the effect size (logFC) of differential gene expression of *FGF14*, *PVALB*, *VGAT* and *GAD67* between controls and schizophrenia patients in post-mortem dorsolateral prefrontal cortex (DLPFC) (BA46). Linear model and empirical Bayes method (Limma) was applied for assessing the differential gene expression of *FGF14* and its co-expression genes, including *PVALB*, *GAD67* and *VGAT*, in two independent data sets (GSE21138 and GSE12649) deposited in NCBI GEO. LogFC>0 suggest decreased gene expression in patients with schizophrenia. The forest plot was created by R rmeta package. VGAT, vesicular gamma-amino-butyric acid transporter.^61, 62, 63, 64^

To provide translational value to our studies, we examined large transcriptomic data sets from schizophrenia post-mortem tissues deposited in the NCBI Gene Expression Omnibus, seeking genes whose expression might covary with that of *FGF14*. Through the SEEK-based gene co-expression search engine with built-in functional annotation and pathway enrichment by Gene Ontology terms (Supplementary Table 2) and by KEGG ortholog analysis, we found that *FGF14* was enriched within the ‘GABAergic synapse' pathway (Supplementary Table 1) and its expression profile correlated with that of *PVALB* (*P*=0.004 in hippocampus; *P*=0.0059 in prefrontal cortex), *GAD67* (*P*=0.0009 in hippocampus; *P*=0.0003 in prefrontal cortex) and *VGAT* (*P*=0.04 in hippocampus; *P*=0.069 in prefrontal cortex) (Supplementary Table 3) in tissue/disease-specific conditions including schizophrenia. Western blot analysis confirmed a significant reduction of GAD67 and VGAT protein expression in the hippocampus (Figures 2g and h) as well as in the prefrontal cortex (Supplementary Fig.5A, B) in *Fgf14*^−/−^ mice compared with controls, providing further correlations between our mouse model and human studies. We subsequently analyzed expression of the *FGF14*, *PVALB*, *GAD67* and *VGAT* genes and their correlations in two schizophrenia-enriched data sets and matched controls from the dorsal lateral prefrontal cortex.^60, 61^ Both data sets showed a significant decreased expression of *FGF14*, *PVALB*, *GAD67* and *VGAT* (Figure 5a and Supplementary Figure 6), and a highly significant correlation between *FGF14*, *PVALB*, *GAD67* and *VGAT* was found in all samples and in schizophrenia alone with the largest effect size in schizophrenia and controls for *GAD67* (Figure 5b). The top enriched pathway such as ‘GABAergic synapse' was also confirmed by another pathway enrichment tool, GeneMANIA and the GABAergic gene cluster was identified by STRING as showed in Supplementary Table 4 and Supplementary Figure 8.

## Discussion

Here we provide new evidence for FGF14 in maintaining GABAergic activity in the CA1 hippocampal region, an area critical for cognitive function.^65^ Genetic deletion of *Fgf14* leads to a decrease in the number of PV interneurons and in the expression level of the presynaptic GABAergic markers GAD67 and VGAT. These changes are associated with reduced inhibitory tone of pyramidal neurons, decreased gamma frequency oscillations and deficits in working memory. Bioinformatics analysis from human transcriptomics identified FGF14 as a component of GABAergic synaptic signaling and revealed a correlated decrease in *FGF14*, *PVALB*, *GAD67* and *VGAT* gene expression in schizophrenia post-mortem tissues compared with matched controls. These results provide a new mechanistic role for FGF14, an emerging neuropsychiatric disease-associated gene^17, 18, 19, 20, 21, 22, 23, 24, 25, 26, 27^ in the context of human brain disorders.

The identification of FGF14 immunoreactivity at the AIS of CA1 PV interneurons suggests this protein contributes to interneuron structural and functional diversity^66^ and as such might be part of the repertoire of signaling molecules dictating the cell cardinal and definitive specifications of PV neurons in the neural circuitry.^1, 67^ The cell type and sublayer-specific loss of PV neurons observed upon *Fgf14* deletion confirms this hypothesis and supports the notion that FGF14 is indispensable for the development, maintenance and/or survival of PV interneurons in the CA1 region. Our post-mortem human tissue study identifies a significant correlation between the *Fgf14* and *PVALB* genes, which might contribute to the loss of PV interneurons observed in *Fgf14*^−/−^ mice.

We also found that the expression level of GAD67 and VGAT, two well-characterized disease-associated proteins critical for GABA synthesis and differentiation in addition to synaptogenesis of PV neurons,^68, 69, 70, 71^ are decreased in PV-positive somas and at inhibitory presynaptic terminals in *Fgf14*^−/−^ animals. This phenotype is consistent with a diminished total pool of the two proteins, which might result from covariance of *FGF14*, *GAD67* and *VGAT* at expression level as suggested by our bioinformatics analysis from schizophrenia samples.

Whether loss of PV neurons and deficits in GABAergic markers in *Fgf14*^−/−^ brains occur through causative loops or are separate coincidental events remains to be determined. Evidence exists for two separate functions of FGF14 as a regulator of intrinsic excitability at the AIS^30, 33, 34, 37, 38, 42, 43^ and a presynaptic organizer.^40, 41^ Thus, phenotypes observed in *Fgf14*^−/−^ hippocampi might arise from disruption of two independent functions of FGF14: one impairing intrinsic firing of PV neurons, causing cell death, arrested development and/or aberrant circuitry integration, and one disrupting the presynaptic GABA machinery (synthesis and loading) via downregulation of selective markers (that is, GAD67 and VGAT). Convergence of these disrupted functions might have fatal consequences for the final specifications and circuitry integration in PV neurons in the CA1 region. In an *Fgf14* null condition, these uncompensated functions could be aggravated by concomitant loss of excitatory inputs, leading to a global remodeling of PV innervation fields,^69^ shifting cortical networks into a high-PV status with reduced plasticity.^68^

Given that the majority of GABAergic inputs in the CA1 area arise from PV interneurons, and that GAD67 and VGAT are essential for the synthesis and loading of GABA at presynaptic terminals, we posited that *Fgf14* genetic deletion might impair hippocampal inhibitory transmission. Consistently, the distribution of sIPSC frequency (and amplitude) in *Fgf14*^−/−^ CA1 pyramidal neurons was found populated by longer inter-event intervals compared with *Fgf14*^*+/+*^ suggesting that in *Fgf14*^−/−^ mice the remaining inhibitory neurons might fire less than in control animals. sIPSCs are action potential-driven synaptic events and as such represent a compound readout of the firing status of presynaptic interneurons and the neurotransmitter release machinery. To examine GABA release mechanisms independent of interneuron spontaneous firing, we isolated mIPSCs. The distributions of frequency and amplitude of mIPSCs in *Fgf14*^−/−^ mice were shifted with loss in short inter-event intervals and in smallest and largest amplitude events, respectively. These results highlight a combination of pre- and postsynaptic deficits in GABAergic transmission possibly induced by lower quantum content, probability of vesicle release or reduced number of vesicle, which might arise from structural and/or functional loss of a subset of inhibitory terminals. The lack in change of rise and decay time in the *Fgf14*^−/−^ mice argues against significant changes in the mechanism of diffusion of GABA across the synaptic cleft, or in the composition of postsynaptic ionotropic GABA receptors. However, high-resolution structural analysis is required for confirmation.

In cortical areas loss in PV neuron function and changes in GABAergic activity can desynchronize the E/I network leading to reduced gamma oscillations, a phenotype associated with schizophrenia and other psychiatric disorders.^1, 3, 72^ *In vivo* electroencephalogram local field potential recordings in the CA1 region revealed that in *Fgf14*^−/−^ animals the total, slow and fast, gamma band power was suppressed compared with control mice. These temporally segregated gamma oscillations reflect information carried by CA3 Schaffer collaterals and perforant path inputs, respectively.^73, 74, 75^ Thus, a reduction in both slow and fast gamma implies that both intra- and extra-hippocampal synaptic inputs might be compromised, possibly reflecting more widespread anomalies in *Fgf14*^−/−^ brains, arising from the prefrontal cortex–thalamic–hippocampal loop.^76^ At the behavioral level, we show that *Fgf14*^−/−^ animals exhibit impaired spatial working memory, complementing the array of deficits commonly found in psychiatric disorders associated with cognitive impairment.^59^

Human transcriptomics data confirmed functional clustering of *FGF14* with GABAergic signaling and identified a highly correlated decrease of *FGF14*, *PVALB*, *GAD67* and *VGAT* in schizophrenia post-mortem tissues, indicating possible genetic co-regulation of these genes. Thus, diminished expression of FGF14 in humans might be a risk factor for complex brain diseases associated with cognitive impairment such as schizophrenia. These findings extend the original studies linking the dominant negative *FGF14*^*F145S*^ missense mutation to the inherited, rare disorder SCA27 to a much broader set of human brain diseases.

The range of phenotypes from molecular to behavioral observed in *Fgf14*^−/−^ mice along with our corroborating human studies lay the groundwork for new mechanistic hypotheses on the biology and potential risk factors of cognitive impairment in schizophrenia and other complex brain disorders associated with E/I tone imbalance and disrupted development and plasticity of GABAergic signaling.^77^ These findings further strengthen the emerging role of the AIS and its molecular components in the biology of diseases such as schizophrenia, bipolar disorder and depression.^42^

## Notes

HYM has served as a consultant for Forum, Lundbeck, Reviva and Sunovion; he owns stocks in Acadia Pharmaceuticals, GlaxoSmithKline and SureGene. The remaining authors declare no conflict of interest.

## References

[bib1] Lewis DA, Curley AA, Glausier JR, Volk DW. Cortical parvalbumin interneurons and cognitive dysfunction in schizophrenia. Trends Neurosci 2012; 35: 57–67. 22154068 10.1016/j.tins.2011.10.004 PMC3253230

[bib2] Nissen S, Liang S, Shehktman T, Kelsoe JR, Bipolar Genome S, Greenwood TA et al. Evidence for association of bipolar disorder to haplotypes in the 22q12.3 region near the genes stargazin, IFT27 and parvalbumin. Am J Med Genet B Neuropsychiatr Genet 2012; 159B: 941–950. 23038240 10.1002/ajmg.b.32099 PMC3665332

[bib3] Yizhar O, Fenno LE, Prigge M, Schneider F, Davidson TJ, O'Shea DJ et al. Neocortical excitation/inhibition balance in information processing and social dysfunction. Nature 2011; 477: 171–178. 21796121 10.1038/nature10360 PMC4155501

[bib4] Curley AA, Eggan SM, Lazarus MS, Huang ZJ, Volk DW, Lewis DA. Role of glutamic acid decarboxylase 67 in regulating cortical parvalbumin and GABA membrane transporter 1 expression: implications for schizophrenia. Neurobiol Dis 2013; 50: 179–186. 23103418 10.1016/j.nbd.2012.10.018 PMC3534919

[bib5] Eggan SM, Lazarus MS, Stoyak SR, Volk DW, Glausier JR, Huang ZJ et al. Cortical glutamic acid decarboxylase 67 deficiency results in lower cannabinoid 1 receptor messenger RNA expression: implications for schizophrenia. Biol Psychiatry 2012; 71: 114–119. 22036037 10.1016/j.biopsych.2011.09.014 PMC3237751

[bib6] Hashimoto T, Arion D, Unger T, Maldonado-Aviles JG, Morris HM, Volk DW et al. Alterations in GABA-related transcriptome in the dorsolateral prefrontal cortex of subjects with schizophrenia. Mol Psychiatry 2008; 13: 147–161. 17471287 10.1038/sj.mp.4002011 PMC2882638

[bib7] Lewis DA, Hashimoto T, Morris HM. Cell and receptor type-specific alterations in markers of GABA neurotransmission in the prefrontal cortex of subjects with schizophrenia. Neurotox Res 2008; 14: 237–248. 19073429 10.1007/BF03033813 PMC2884395

[bib8] Lewis DA, Hashimoto T, Volk DW. Cortical inhibitory neurons and schizophrenia. Nat Rev Neurosci 2005; 6: 312–324. 15803162 10.1038/nrn1648

[bib9] Lewis DA, Volk DW, Hashimoto T. Selective alterations in prefrontal cortical GABA neurotransmission in schizophrenia: a novel target for the treatment of working memory dysfunction. Psychopharmacology (Berl) 2004; 174: 143–150. 15205885 10.1007/s00213-003-1673-x

[bib10] Volk DW, Lewis DA. Prefrontal cortical circuits in schizophrenia. Curr Top Behav Neurosci 2010; 4: 485–508. 21312410 10.1007/7854_2010_44

[bib11] Guidotti A, Auta J, Chen Y, Davis JM, Dong E, Gavin DP et al. Epigenetic GABAergic targets in schizophrenia and bipolar disorder. Neuropharmacology 2011; 60: 1007–1016. 21074545 10.1016/j.neuropharm.2010.10.021 PMC4144274

[bib12] Conde F, Lund JS, Jacobowitz DM, Baimbridge KG, Lewis DA. Local circuit neurons immunoreactive for calretinin, calbindin D-28k or parvalbumin in monkey prefrontal cortex: distribution and morphology. J Comp Neurol 1994; 341: 95–116. 8006226 10.1002/cne.903410109

[bib13] Beasley CL, Zhang ZJ, Patten I, Reynolds GP. Selective deficits in prefrontal cortical GABAergic neurons in schizophrenia defined by the presence of calcium-binding proteins. Biol Psychiatry 2002; 52: 708–715. 12372661 10.1016/s0006-3223(02)01360-4

[bib14] Knable MB, Barci BM, Webster MJ, Meador-Woodruff J, Torrey EFStanley Neuropathology C. Molecular abnormalities of the hippocampus in severe psychiatric illness: postmortem findings from the Stanley Neuropathology Consortium. Mol Psychiatry 2004; 9: 544. 10.1038/sj.mp.4001471 14708030

[bib15] Zhang ZJ, Reynolds GP. A selective decrease in the relative density of parvalbumin-immunoreactive neurons in the hippocampus in schizophrenia. Schizophr Res 2002; 55: 1–10. 11955958 10.1016/s0920-9964(01)00188-8

[bib16] Torrey EF, Barci BM, Webster MJ, Bartko JJ, Meador-Woodruff JH, Knable MB. Neurochemical markers for schizophrenia, bipolar disorder, and major depression in postmortem brains. Biol Psychiatry 2005; 57: 252–260. 15691526 10.1016/j.biopsych.2004.10.019

[bib17] Drgon T, Zhang PW, Johnson C, Walther D, Hess J, Nino M et al. Genome wide association for addiction: replicated results and comparisons of two analytic approaches. PLoS One 2010; 5: e8832. 20098672 10.1371/journal.pone.0008832 PMC2809089

[bib18] Jungerius BJ, Hoogendoorn ML, Bakker SC, Van't Slot R, Bardoel AF, Ophoff RA et al. An association screen of myelin-related genes implicates the chromosome 22q11 PIK4CA gene in schizophrenia. Mol Psychiatry 2008; 13: 1060–1068. 17893707 10.1038/sj.mp.4002080

[bib19] Spencer JR, Darbyshire KM, Boucher AA, Kashem MA, Long LE, McGregor IS et al. Novel molecular changes induced by Nrg1 hypomorphism and Nrg1-cannabinoid interaction in adolescence: a hippocampal proteomic study in mice. Front Cell Neurosci 2013; 7: 15. 23447498 10.3389/fncel.2013.00015 PMC3581856

[bib20] Chen HM, DeLong CJ, Bame M, Rajapakse I, Herron TJ, McInnis MG et al. Transcripts involved in calcium signaling and telencephalic neuronal fate are altered in induced pluripotent stem cells from bipolar disorder patients. Transl Psychiatry 2014; 4: e375. 25116795 10.1038/tp.2014.12 PMC3966040

[bib21] Hodgkinson CA, Enoch MA, Srivastava V, Cummins-Oman JS, Ferrier C, Iarikova P et al. Genome-wide association identifies candidate genes that influence the human electroencephalogram. Proc Natl Acad Sci USA 2010; 107: 8695–8700. 20421487 10.1073/pnas.0908134107 PMC2889314

[bib22] Olson H, Shen Y, Avallone J, Sheidley BR, Pinsky R, Bergin AM et al. Copy number variation plays an important role in clinical epilepsy. Ann Neurol 2014; 75: 943–958. 24811917 10.1002/ana.24178 PMC4487364

[bib23] Hu X-L, Cheng X, Cai L, Tan G-H, Xu L, Feng X-Y et al. Conditional deletion of NRSF in forebrain neurons accelerates epileptogenesis in the kindling model. Cerebr Cortex 2011; 21: 2158–2165. 10.1093/cercor/bhq284 21339379

[bib24] Liu Q-R, Drgon T, Johnson C, Walther D, Hess J, Uhl GR. Addiction molecular genetics: 639,401 SNP whole genome association identifies many “cell adhesion” genes. Am J Med Genet 2006; 141B: 918–925. 17099884 10.1002/ajmg.b.30436

[bib25] Hunter AM, Leuchter AF, Power RA, Muthen B, McGrath PJ, Lewis CM et al. A genome-wide association study of a sustained pattern of antidepressant response. J Psychiatr Res 2013; 47: 1157–1165. 23726668 10.1016/j.jpsychires.2013.05.002 PMC3710535

[bib26] Verbeek EC, Bakker IM, Bevova MR, Bochdanovits Z, Rizzu P, Sondervan D et al. A fine-mapping study of 7 top scoring genes from a GWAS for major depressive disorder. PLoS One 2012; 7: e37384. 22649524 10.1371/journal.pone.0037384 PMC3359349

[bib27] Brennand KJ, Simone A, Jou J, Gelboin-Burkhart C, Tran N, Sangar S et al. Modelling schizophrenia using human induced pluripotent stem cells. Nature 2011; 473: 221–225. 21490598 10.1038/nature09915 PMC3392969

[bib28] Brusse E, de Koning I, Maat-Kievit A, Oostra BA, Heutink P, van Swieten JC. Spinocerebellar ataxia associated with a mutation in the fibroblast growth factor 14 gene (SCA27): A new phenotype. Mov Disord 2006; 21: 396–401. 16211615 10.1002/mds.20708

[bib29] Van Swieten JC, Brusse E, De Graaf BM, Krieger E, Van De Graaf R, De Koning I et al. A mutation in the fibroblast growth factor 14 gene is associated with autosomal dominant cerebellar ataxia. Am J Hum Genet 2003; 72: 191–199. 12489043 10.1086/345488 PMC378625

[bib30] Laezza F, Gerber BR, Lou JY, Kozel MA, Hartman H, Craig AM et al. The FGF14(F145S) mutation disrupts the interaction of FGF14 with voltage-gated Na+ channels and impairs neuronal excitability. J Neurosci 2007; 27: 12033–12044. 17978045 10.1523/JNEUROSCI.2282-07.2007 PMC6673376

[bib31] Laezza F, Lampert A, Kozel MA, Gerber BR, Rush AM, Nerbonne JM et al. FGF14 N-terminal splice variants differentially modulate Nav1.2 and Nav1.6-encoded sodium channels. Mol Cell Neurosci 2009; 42: 90–101. 19465131 10.1016/j.mcn.2009.05.007 PMC2832592

[bib32] Goetz R, Dover K, Laezza F, Shtraizent N, Huang X, Tchetchik D et al. Crystal structure of a fibroblast growth factor homologous factor (FHF) defines a conserved surface on FHFs for binding and modulation of voltage-gated sodium channels. J Biol Chem 2009; 284: 17883–17896. 19406745 10.1074/jbc.M109.001842 PMC2719427

[bib33] Shavkunov A, Panova N, Prasai A, Veselenak R, Bourne N, Stoilova-McPhie S et al. Bioluminescence methodology for the detection of protein-protein interactions within the voltage-gated sodium channel macromolecular complex. Assay Drug Dev Technol 2012; 10: 148–160. 22364545 10.1089/adt.2011.413 PMC3362325

[bib34] Shavkunov AS, Wildburger NC, Nenov MN, James TF, Buzhdygan TP, Panova-Elektronova NI et al. The fibroblast growth factor 14: voltage-gated sodium channel complex is a new target of glycogen synthase kinase 3 (GSK3). J Biol Chem 2013; 288: 19370–19385. 23640885 10.1074/jbc.M112.445924 PMC3707642

[bib35] James TF, Nenov MN, Wildburger NC, Lichti CF, Luisi J, Vergara F et al. The Na1.2 channel is regulated by GSK3. Biochim Biophys Acta 2015; 1850: 832–844. 25615535 10.1016/j.bbagen.2015.01.011 PMC4336163

[bib36] Ali SR, Panova N, Stoilova-McPhie S, Laezza F. Protein-protein interactions based drug discovery against the voltage-gated sodium channel. Biophys J 2014; 106: 326a–326aa.

[bib37] Shakkottai VG, Xiao M, Xu L, Wong M, Nerbonne JM, Ornitz DM et al. FGF14 regulates the intrinsic excitability of cerebellar Purkinje neurons. Neurobiol Dis 2009; 33: 81–88. 18930825 10.1016/j.nbd.2008.09.019 PMC2652849

[bib38] Goldfarb M, Schoorlemmer J, Williams A, Diwakar S, Wang Q, Huang X et al. Fibroblast growth factor homologous factors control neuronal excitability through modulation of voltage-gated sodium channels. Neuron 2007; 55: 449–463. 17678857 10.1016/j.neuron.2007.07.006 PMC2974323

[bib39] Bosch MK, Carrasquillo Y, Ransdell JL, Kanakamedala A, Ornitz DM, Nerbonne JM. Intracellular FGF14 (iFGF14) is required for spontaneous and evoked firing in cerebellar Purkinje neurons and for motor coordination and balance. J Neurosci 2015; 35: 6752–6769. 25926453 10.1523/JNEUROSCI.2663-14.2015 PMC4412895

[bib40] Yan H, Pablo JL, Pitt GS. FGF14 regulates presynaptic Ca2+ channels and synaptic transmission. Cell Rep 2013; 4: 66–75. 23831029 10.1016/j.celrep.2013.06.012 PMC3736584

[bib41] Xiao M, Xu L, Laezza F, Yamada K, Feng S, Ornitz DM. Impaired hippocampal synaptic transmission and plasticity in mice lacking fibroblast growth factor 14. Mol Cell Neurosci 2007; 34: 366–377. 17208450 10.1016/j.mcn.2006.11.020

[bib42] Hsu WC, Nilsson CL, Laezza F. Role of the axonal initial segment in psychiatric disorders: function, dysfunction, and intervention. Front Psychiatry 2014; 5: 109. 25191280 10.3389/fpsyt.2014.00109 PMC4139700

[bib43] Hsu WC, Nenov MN, Shavkunov A, Panova N, Zhan M, Laezza F. Identifying a kinase network regulating FGF14:Nav1.6 complex assembly using split-luciferase complementation. PLoS One 2015; 10: e0117246. 25659151 10.1371/journal.pone.0117246 PMC4319734

[bib44] Wozniak DF, Xiao M, Xu L, Yamada KA, Ornitz DM. Impaired spatial learning and defective theta burst induced LTP in mice lacking fibroblast growth factor 14. Neurobiol Dis 2007; 1: 14–26. 10.1016/j.nbd.2006.11.014 PMC2267915 17236779

[bib45] Wang Q, Bardgett ME, Wong M, Wozniak DF, Lou J, McNeil BD et al. Ataxia and paroxysmal dyskinesia in mice lacking axonally transported FGF14. Neuron 2002; 35: 25–38. 12123606 10.1016/s0896-6273(02)00744-4

[bib46] Shah A, Lodge DJ. A loss of hippocampal perineuronal nets produces deficits in dopamine system function: relevance to the positive symptoms of schizophrenia. Transl Psychiatry 2013; 3: e215. 23321812 10.1038/tp.2012.145 PMC3566725

[bib47] Boley AM, Perez SM, Lodge DJ. A fundamental role for hippocampal parvalbumin in the dopamine hyperfunction associated with schizophrenia. Schizophr Res 2014; 157: 238–243. 24888524 10.1016/j.schres.2014.05.005 PMC4099272

[bib48] Alshammari MA, Alshammari TK, Laezza F. Improved methods for fluorescence microscopy detection of macromolecules at the axon initial segment. Front Cell Neurosci 2016; 10: 5. 26909021 10.3389/fncel.2016.00005 PMC4754416

[bib49] Alshammari MA, Alshammari TK, Nenov MN, Scala F, Laezza F. Fibroblast growth factor 14 modulates the neurogenesis of granule neurons in the adult dentate gyrus. Mol Neurobiol 2015. 10.1007/s12035-015-9568-5 PMC4916041 26687232

[bib50] Tempia F, Hoxha E, Negro G, Alshammari MA, Alshammari T, Panova-Elektronova N et al. Parallel fiber to Purkinje cell synaptic impairment in a mouse model of spinocerebellar ataxia type 27. Front Cell Neurosci 2015; 9: 205. 26089778 10.3389/fncel.2015.00205 PMC4455242

[bib51] Nenov MN, Laezza F, Haidacher SJ, Zhao Y, Sadygov RG, Starkey JM et al. Cognitive enhancing treatment with a PPARgamma agonist normalizes dentate granule cell presynaptic function in Tg2576 APP mice. J Neurosci 2014; 34: 1028–1036. 24431460 10.1523/JNEUROSCI.3413-13.2014 PMC3891946

[bib52] Nenov MN, Tempia FMP, Denner L, Dineley KT, Laezza F. Impaired firing properties of dentate granule neurons in an Alzheimer's disease animal model are rescued by PPARgamma agonism. J Neurophysiol 2014; 113: 1712–1726. 25540218 10.1152/jn.00419.2014 PMC4359997

[bib53] Cambiaghi M, Cursi M, Magri L, Castoldi V, Comi G, Minicucci F et al. Behavioural and EEG effects of chronic rapamycin treatment in a mouse model of tuberous sclerosis complex. Neuropharmacology 2013; 67: 1–7. 23159330 10.1016/j.neuropharm.2012.11.003

[bib54] Satoh Y, Endo S, Ikeda T, Yamada K, Ito M, Kuroki M et al. Extracellular signal-regulated kinase 2 (ERK2) knockdown mice show deficits in long-term memory; ERK2 has a specific function in learning and memory. J Neurosci 2007; 27: 10765–10776. 17913910 10.1523/JNEUROSCI.0117-07.2007 PMC6672813

[bib55] Zierhut KC, Grassmann R, Kaufmann J, Steiner J, Bogerts B, Schiltz K. Hippocampal CA1 deformity is related to symptom severity and antipsychotic dosage in schizophrenia. Brain 2013; 136(Pt 3): 804–814. 23388407 10.1093/brain/aws335

[bib56] Sawada K, Barr AM, Nakamura M, Arima K, Young CE, Dwork AJ et al. Hippocampal complexin proteins and cognitive dysfunction in schizophrenia. Arch Gen Psychiatry 2005; 62: 263–272. 15753239 10.1001/archpsyc.62.3.263

[bib57] Sawada K, Young CE, Barr AM, Longworth K, Takahashi S, Arango V et al. Altered immunoreactivity of complexin protein in prefrontal cortex in severe mental illness. Mol Psychiatry 2002; 7: 484–492. 12082566 10.1038/sj.mp.4000978

[bib58] Hoftman GD, Volk DW, Bazmi HH, Li S, Sampson AR, Lewis DA. Altered cortical expression of GABA-related genes in schizophrenia: illness progression vs developmental disturbance. Schizophr Bull 2015; 41: 180–191. 24361861 10.1093/schbul/sbt178 PMC4266281

[bib59] Dudchenko PA, Talpos J, Young J, Baxter MG. Animal models of working memory: A review of tasks that might be used in screening drug treatments for the memory impairments found in schizophrenia. Neurosci Biobehav Rev 2013; 37: 2111–2124. 22464948 10.1016/j.neubiorev.2012.03.003

[bib60] Narayan S, Tang B, Head SR, Gilmartin TJ, Sutcliffe JG, Dean B et al. Molecular profiles of schizophrenia in the CNS at different stages of illness. Brain Res 2008; 1239: 235–248. 18778695 10.1016/j.brainres.2008.08.023 PMC2783475

[bib61] Iwamoto K, Bundo M, Kato T. Altered expression of mitochondria-related genes in postmortem brains of patients with bipolar disorder or schizophrenia, as revealed by large-scale DNA microarray analysis. Hum Mol Genet 2005; 14: 241–253. 15563509 10.1093/hmg/ddi022

[bib62] Jimenez D, Labate D, Kakadiaris IA, Papadakis M. Improved automatic centerline tracing for dendritic and axonal structures. Neuroinformatics 2014; 13: 227–244. 10.1007/s12021-014-9256-z 25433514

[bib63] Satoh Y, Endo S, Ikeda T, Yamada K, Ito M, Kuroki M et al. Extracellular signal-regulated kinase 2 (ERK2) knockdown mice show deficits in long-term memory; ERK2 has a specific function in learning and memory. J Neurosci 2007; 27: 10765–10776. 17913910 10.1523/JNEUROSCI.0117-07.2007 PMC6672813

[bib64] Narayan S, Tang B, Head SR, Gilmartin TJ, Sutcliffe JG, Dean B et al. Molecular profiles of schizophrenia in the CNS at different stages of illness. Brain Res 2008; 1239: 235–248. 18778695 10.1016/j.brainres.2008.08.023 PMC2783475

[bib65] Godsil BP, Kiss JP, Spedding M, Jay TM. The hippocampal-prefrontal pathway: the weak link in psychiatric disorders? Eur Neuropsychopharmacol 2013; 23: 1165–1181. 23332457 10.1016/j.euroneuro.2012.10.018

[bib66] Chand AN, Galliano E, Chesters RA, Grubb MS. A distinct subtype of dopaminergic interneuron displays inverted structural plasticity at the axon initial segment. J Neurosci 2015; 35: 1573–1590. 25632134 10.1523/JNEUROSCI.3515-14.2015 PMC4308603

[bib67] Klausberger T, Somogyi P. Neuronal diversity and temporal dynamics: the unity of hippocampal circuit operations. Science 2008; 321: 53–57. 18599766 10.1126/science.1149381 PMC4487503

[bib68] Donato F, Rompani SB, Caroni P. Parvalbumin-expressing basket-cell network plasticity induced by experience regulates adult learning. Nature 2013; 504: 272–276. 24336286 10.1038/nature12866

[bib69] Chattopadhyaya B, Di Cristo G, Wu CZ, Knott G, Kuhlman S, Fu Y et al. GAD67-mediated GABA synthesis and signaling regulate inhibitory synaptic innervation in the visual cortex. Neuron 2007; 54: 889–903. 17582330 10.1016/j.neuron.2007.05.015 PMC2077924

[bib70] Chattopadhyaya B, Di Cristo G, Higashiyama H, Knott GW, Kuhlman SJ, Welker E et al. Experience and activity-dependent maturation of perisomatic GABAergic innervation in primary visual cortex during a postnatal critical period. J Neurosci 2004; 24: 9598–9611. 15509747 10.1523/JNEUROSCI.1851-04.2004 PMC6730138

[bib71] Di Cristo G, Wu C, Chattopadhyaya B, Ango F, Knott G, Welker E et al. Subcellular domain-restricted GABAergic innervation in primary visual cortex in the absence of sensory and thalamic inputs. Nat Neurosci 2004; 7: 1184–1186. 15475951 10.1038/nn1334

[bib72] Craig MT, McBain CJ. Fast gamma oscillations are generated intrinsically in CA1 without the involvement of fast-spiking basket cells. J Neurosci 2015; 35: 3616–3624. 25716860 10.1523/JNEUROSCI.4166-14.2015 PMC4339363

[bib73] Csicsvari J, Jamieson B, Wise KD, Buzsaki G. Mechanisms of gamma oscillations in the hippocampus of the behaving rat. Neuron 2003; 37: 311–322. 12546825 10.1016/s0896-6273(02)01169-8

[bib74] Yamamoto J, Suh J, Takeuchi D, Tonegawa S. Successful execution of working memory linked to synchronized high-frequency gamma oscillations. Cell 2014; 157: 845–857. 24768692 10.1016/j.cell.2014.04.009

[bib75] Colgin LL, Denninger T, Fyhn M, Hafting T, Bonnevie T, Jensen O et al. Frequency of gamma oscillations routes flow of information in the hippocampus. Nature 2009; 462: 353–357. 19924214 10.1038/nature08573

[bib76] Lisman JE, Pi HJ, Zhang Y, Otmakhova NA. A thalamo-hippocampal-ventral tegmental area loop may produce the positive feedback that underlies the psychotic break in schizophrenia. Biol Psychiatry 2010; 68: 17–24. 20553749 10.1016/j.biopsych.2010.04.007 PMC3507433

[bib77] Turner CA, Watson SJ, Akil H. The fibroblast growth factor family: neuromodulation of affective behavior. Neuron 2012; 76: 160–174. 23040813 10.1016/j.neuron.2012.08.037 PMC3476848

