# Supplementary figures and images for: Genetic deletion of fibroblast growth factor 14 recapitulates phenotypic alterations underlying cognitive impairment associated with schizophrenia
Source: Transl Psychiatry. 2016 May 10;6(5):e806–. doi: 10.1038/tp.2016.66 (PMC5070049; doi:10.1038/tp.2016.66)

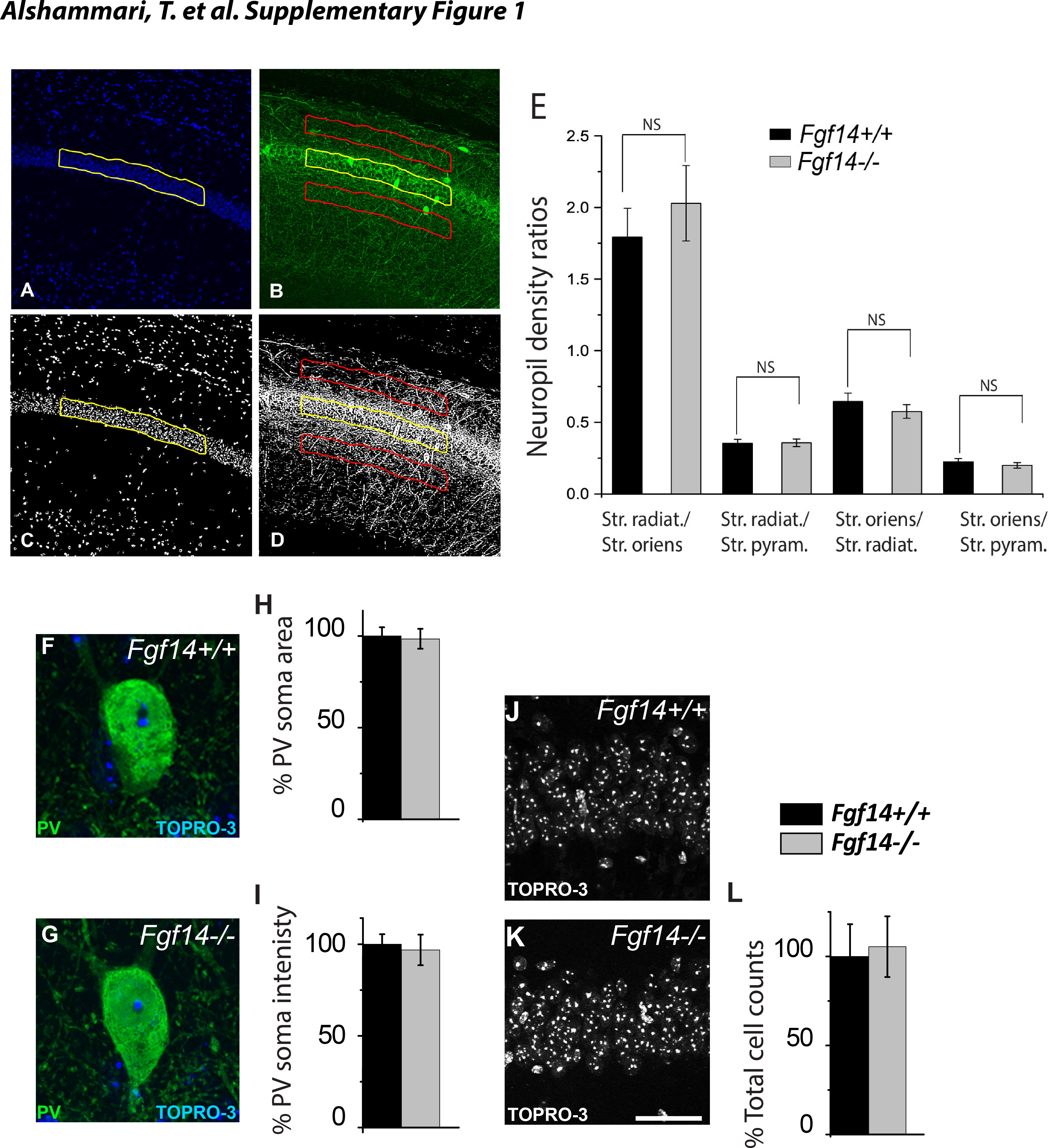

Supplement: Supplementary Figure 1 [file tp201666x1.tif]

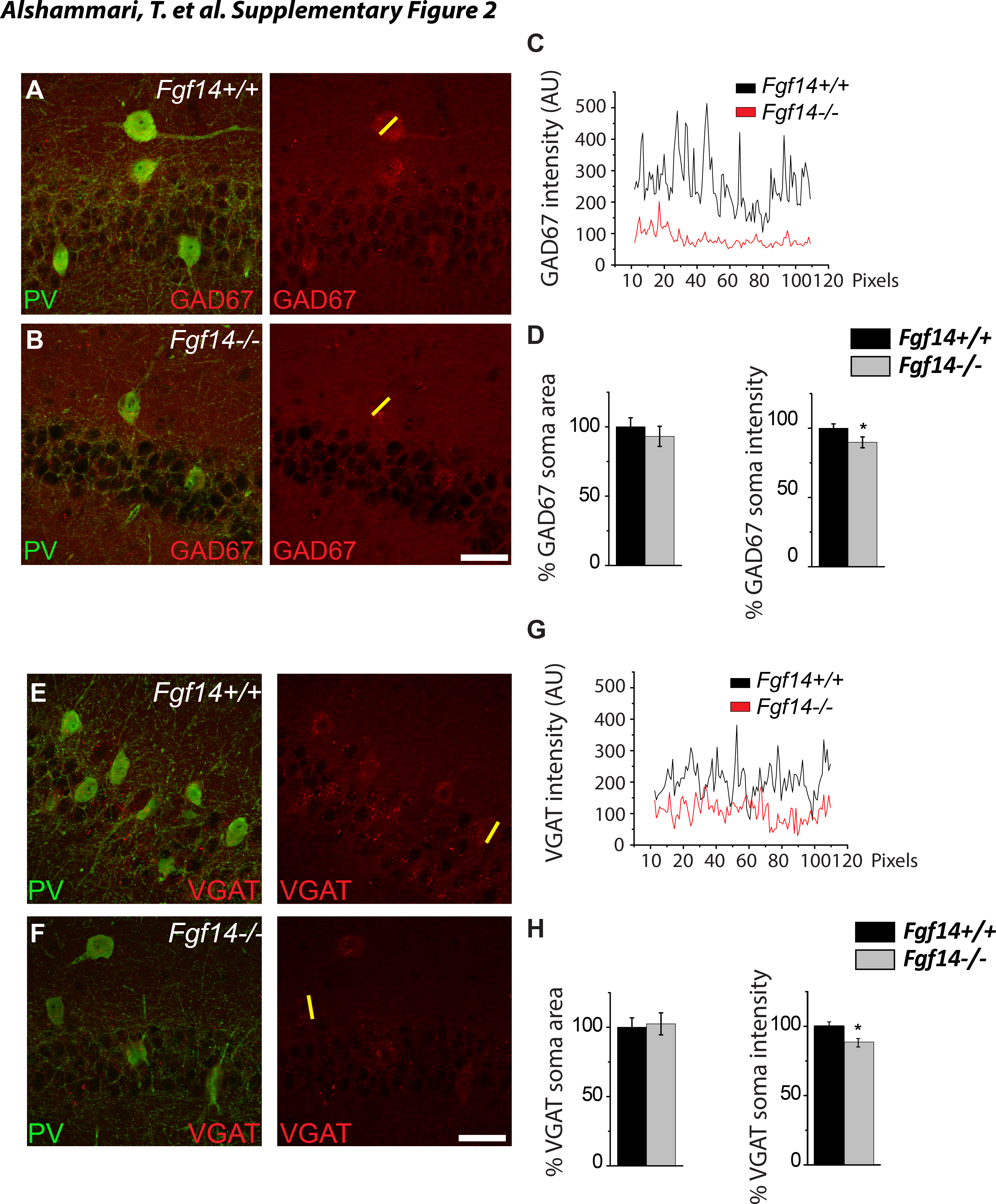

Supplement: Supplementary Figure 2 [file tp201666x2.tif]

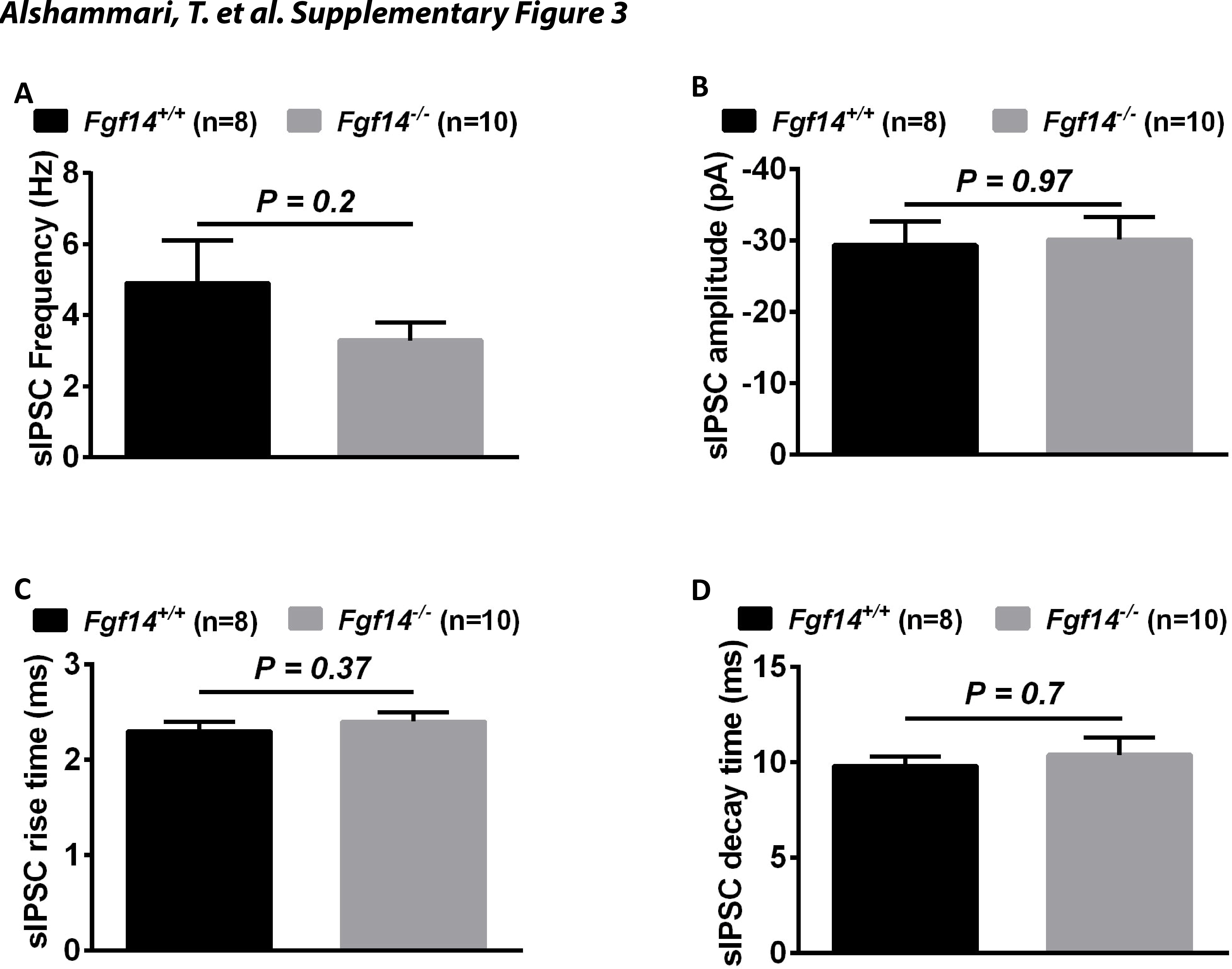

Supplement: Supplementary Figure 3 [file tp201666x3.tif]

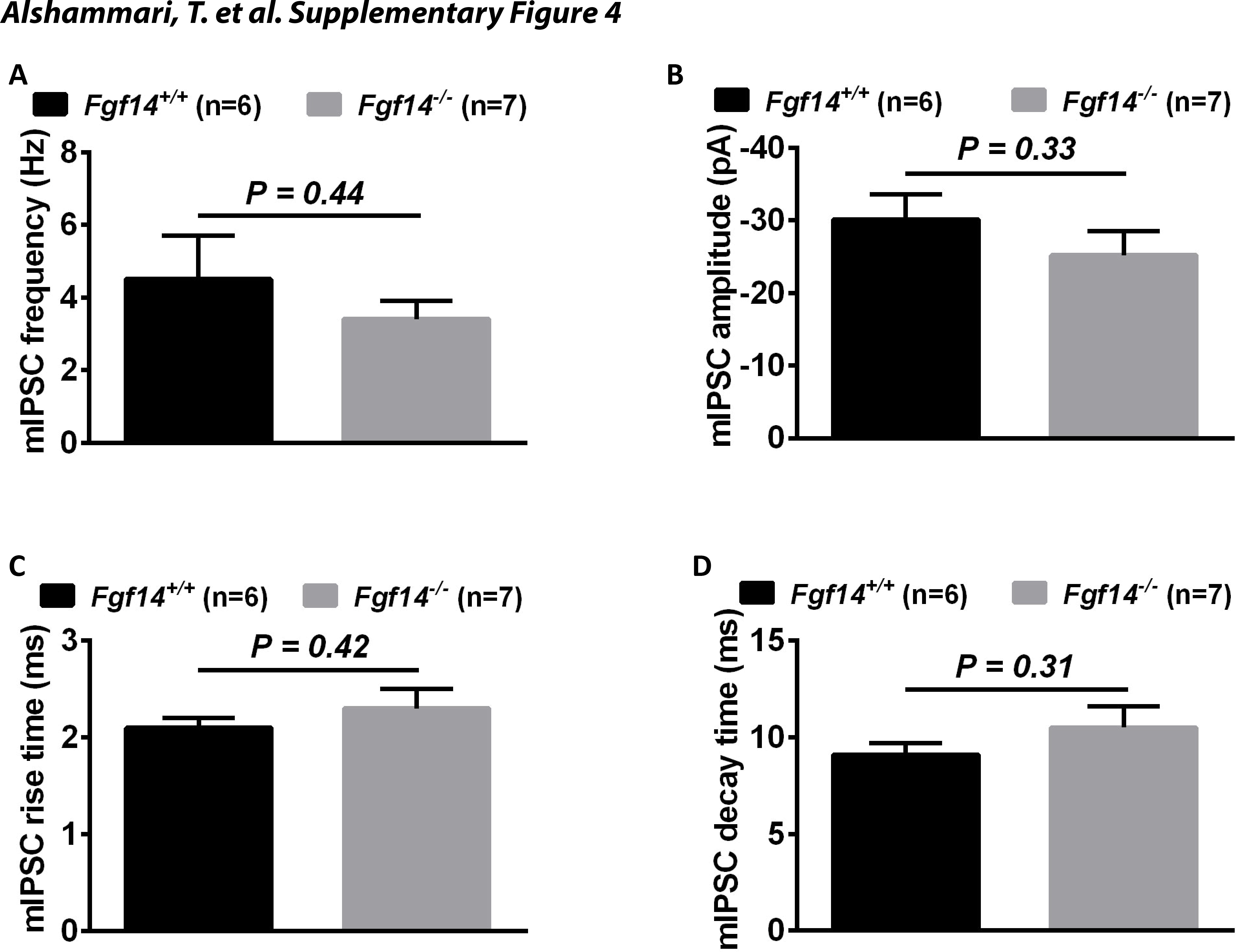

Supplement: Supplementary Figure 4 [file tp201666x4.tif]

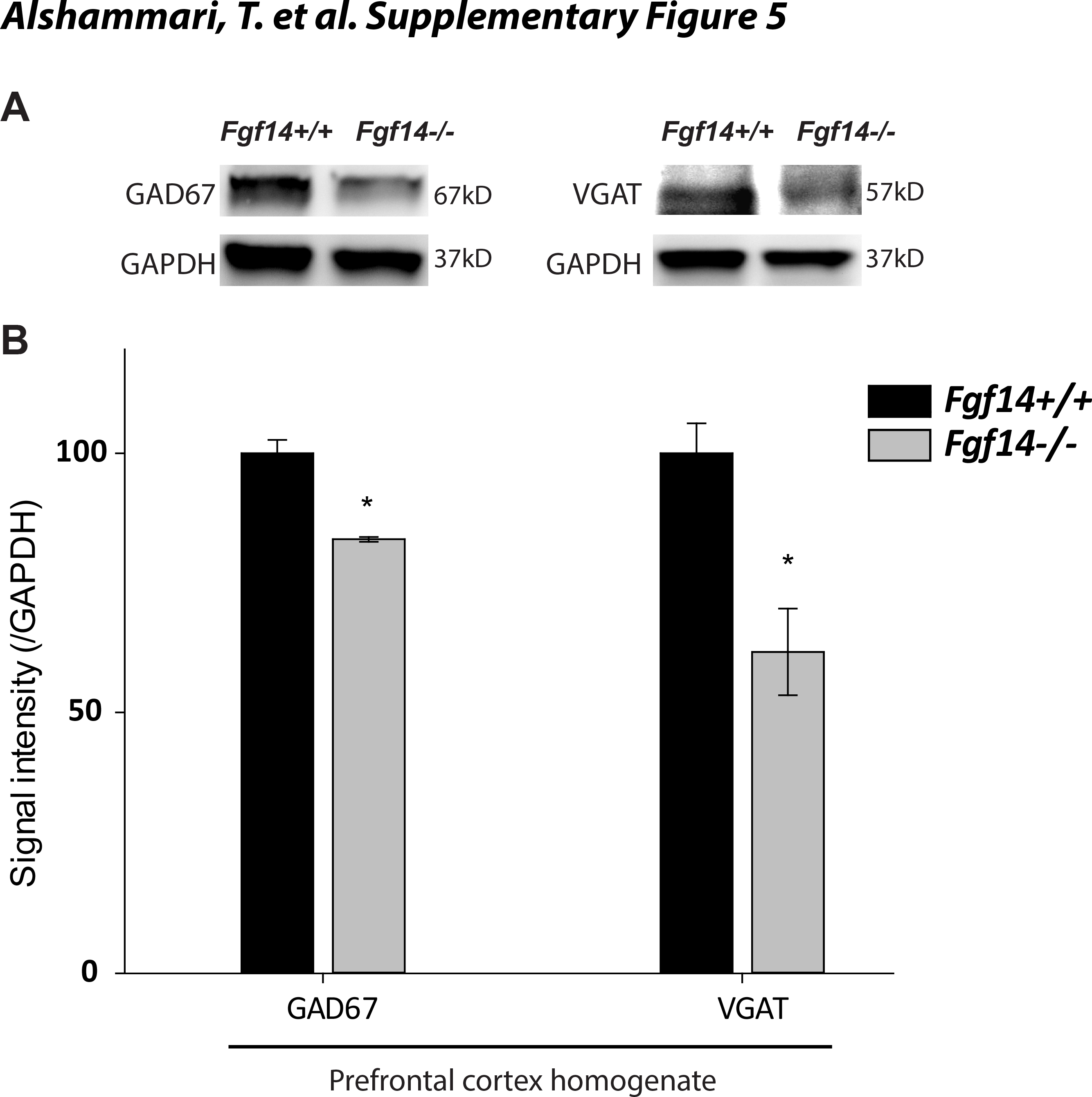

Supplement: Supplementary Figure 5 [file tp201666x5.tif]

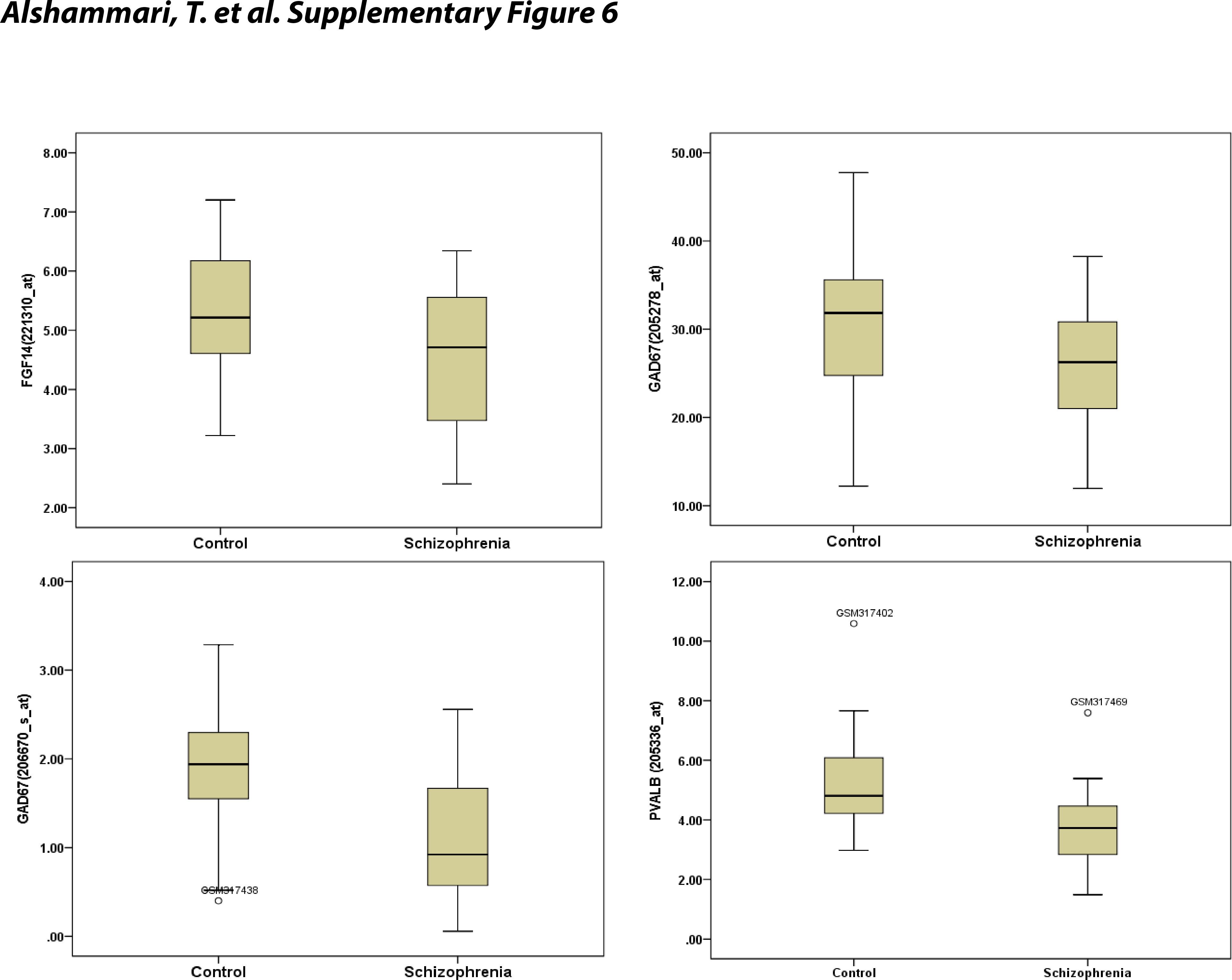

Supplement: Supplementary Figure 6 [file tp201666x6.tif]

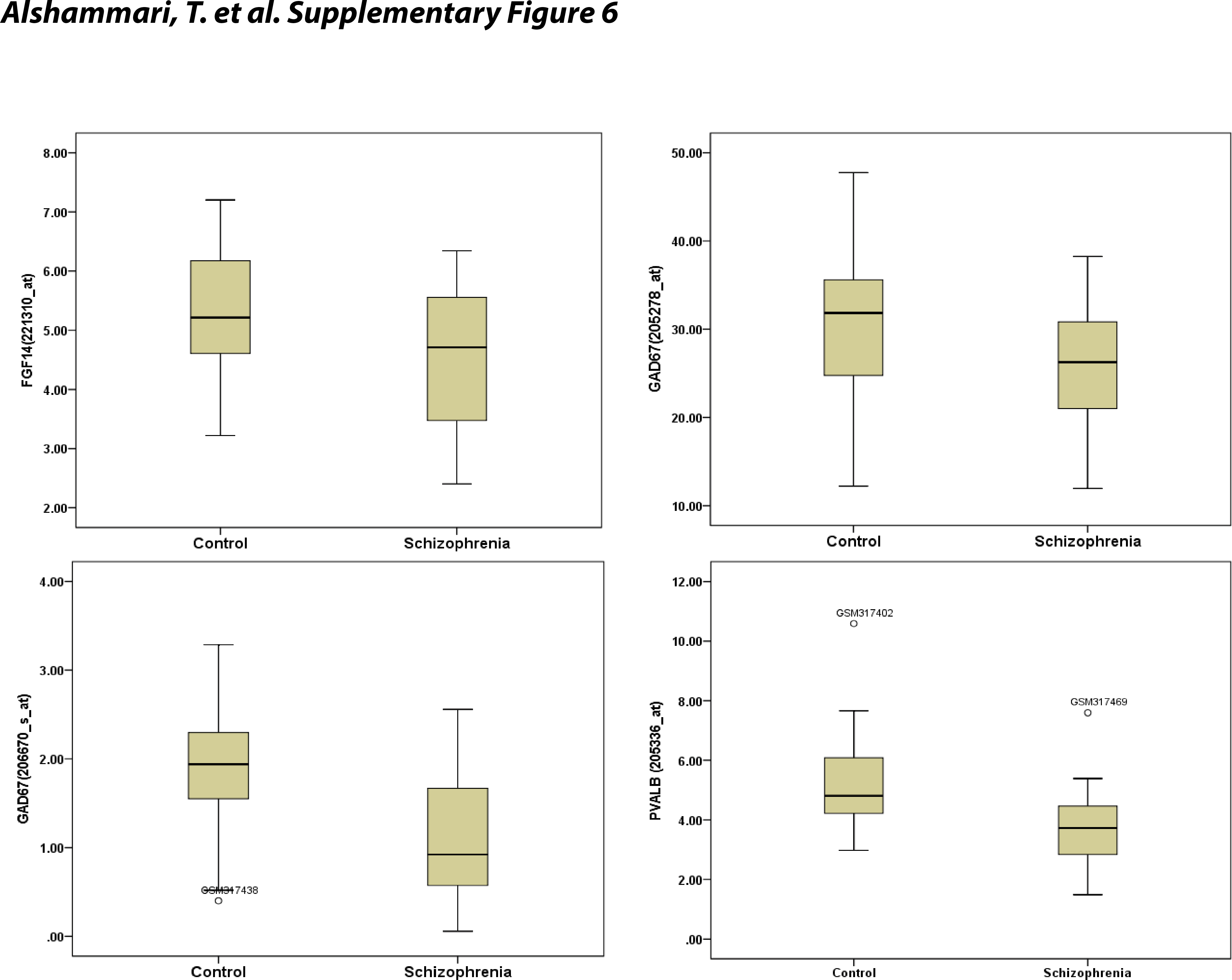

Supplement: Supplementary Figure 7 [file tp201666x7.tif]

A

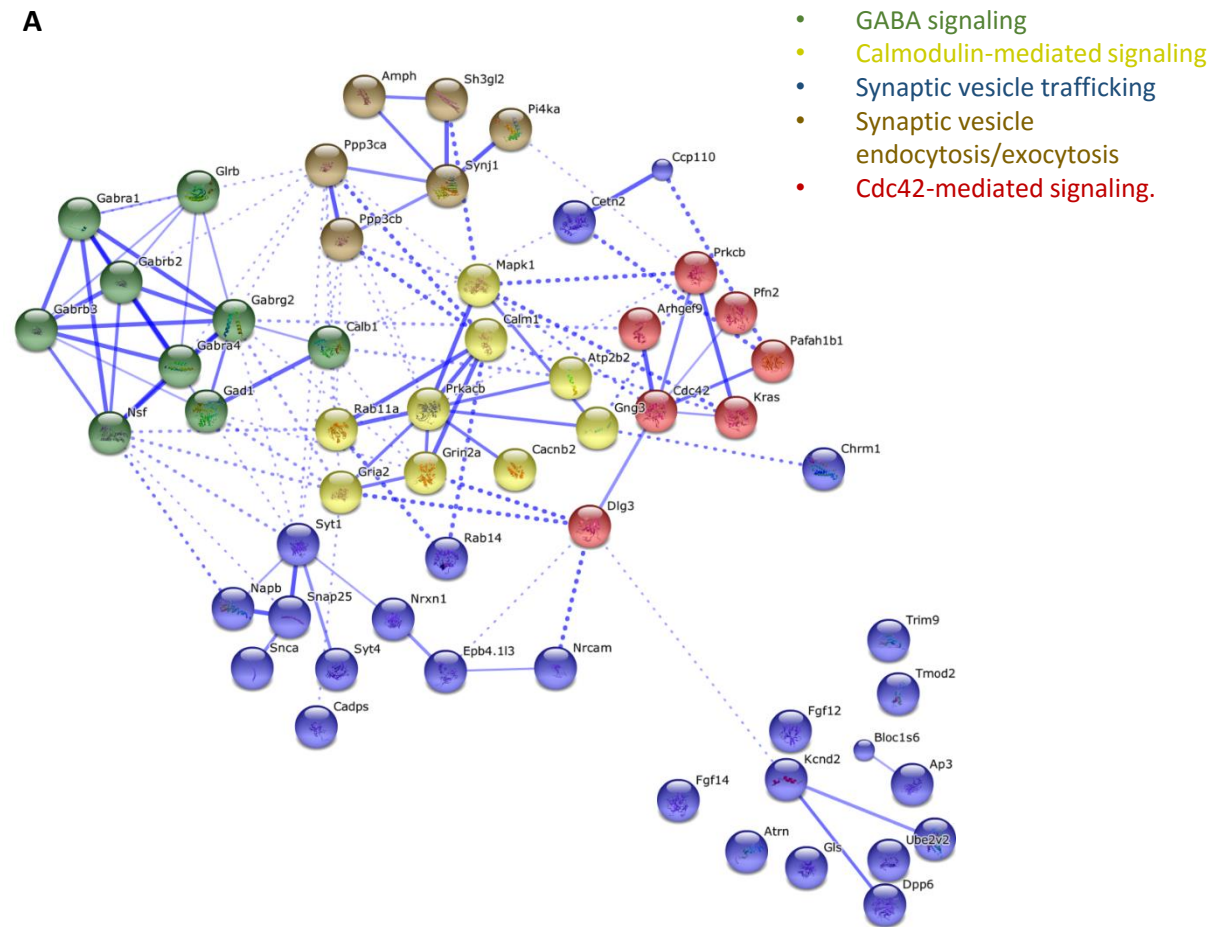

B

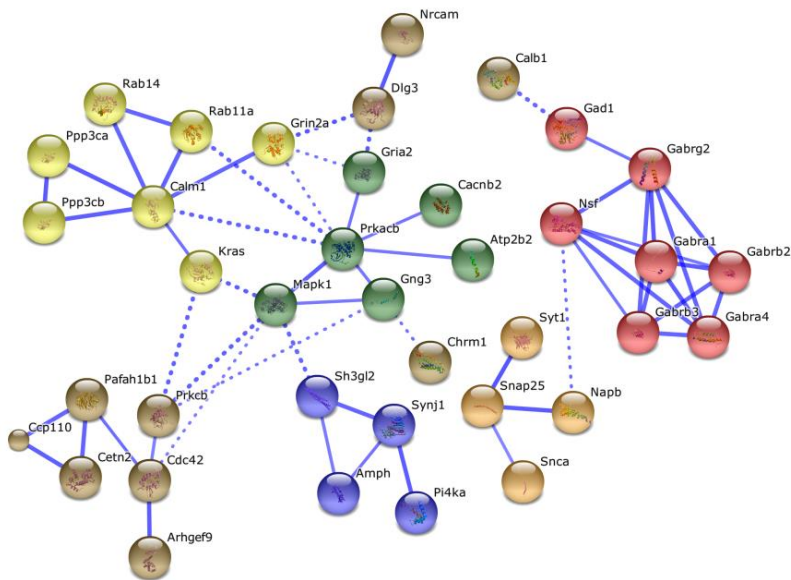

Supplement: Supplementary Figure 8 [file tp201666x8.pdf]

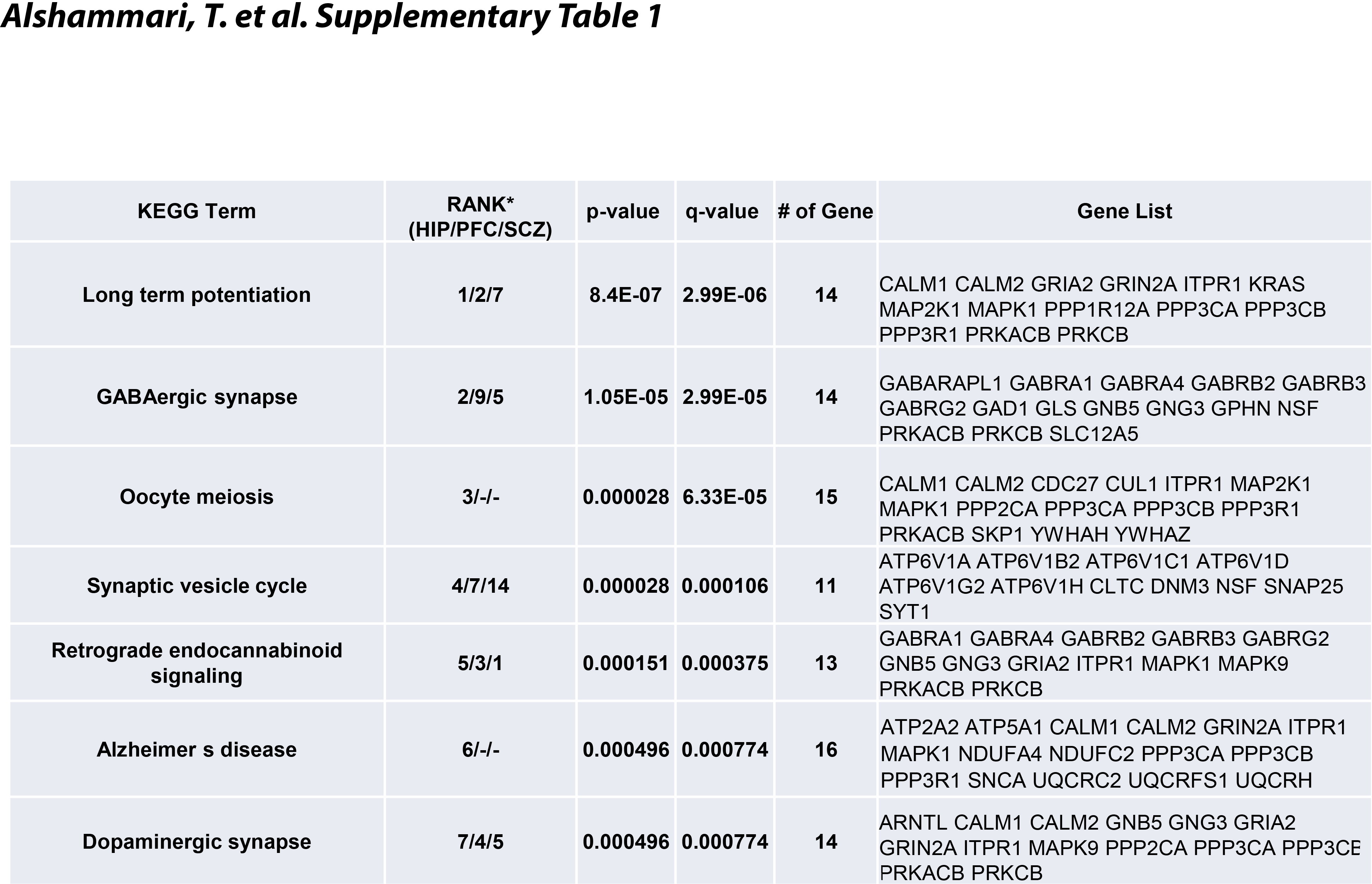

Supplement: Supplementary Table 1 [file tp201666x9.tif]

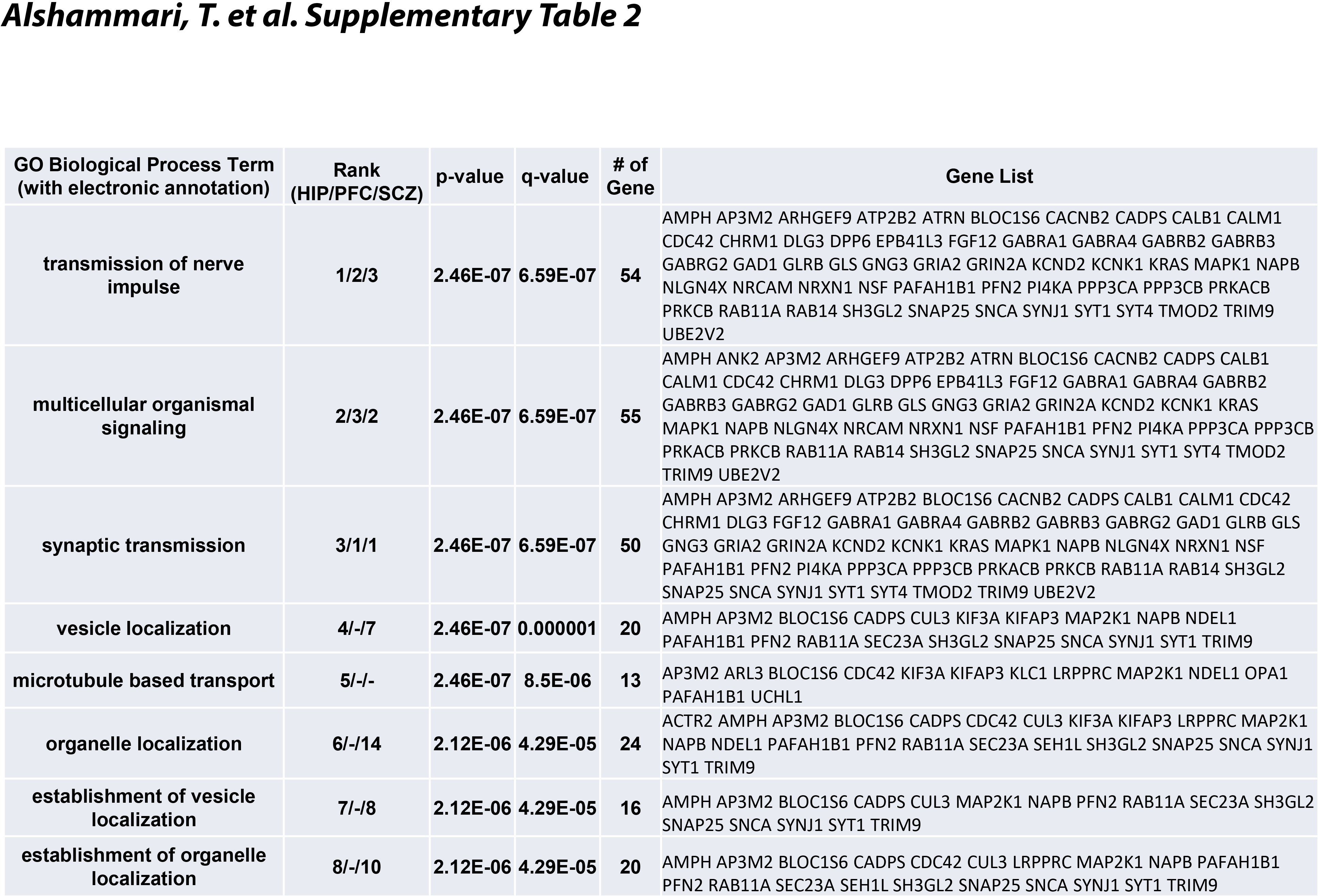

Supplement: Supplementary Table 2 [file tp201666x10.tif]

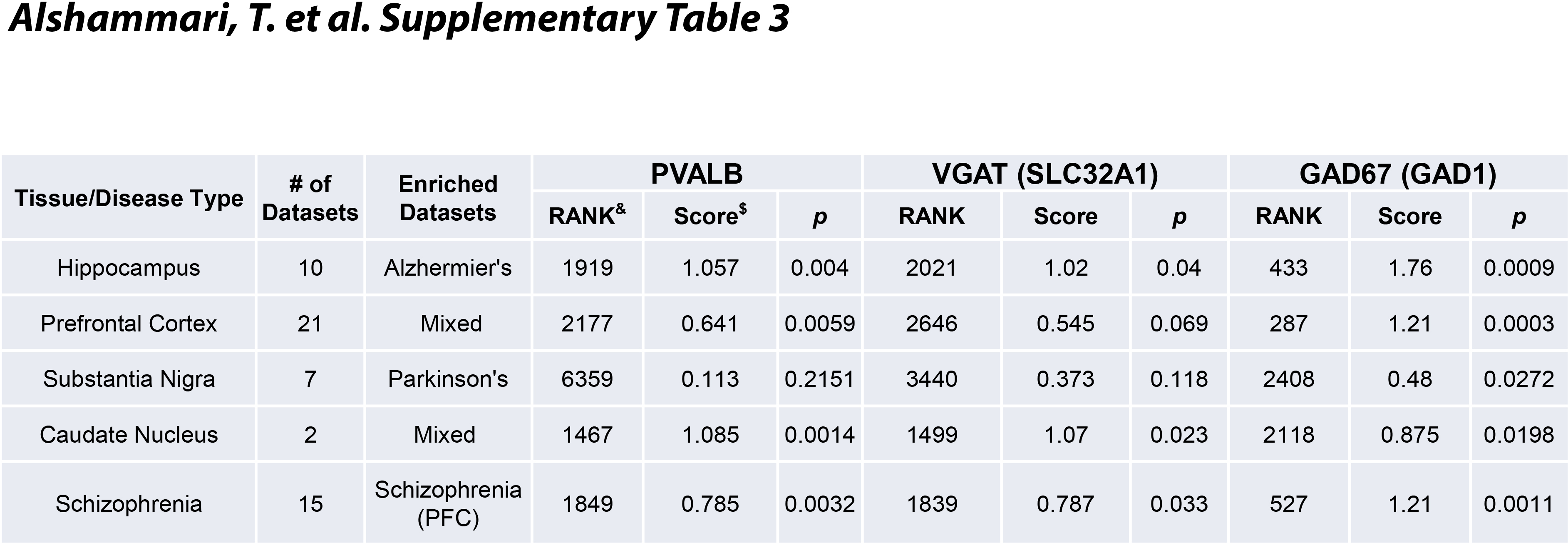

Supplement: Supplementary Table 3 [file tp201666x11.tif]

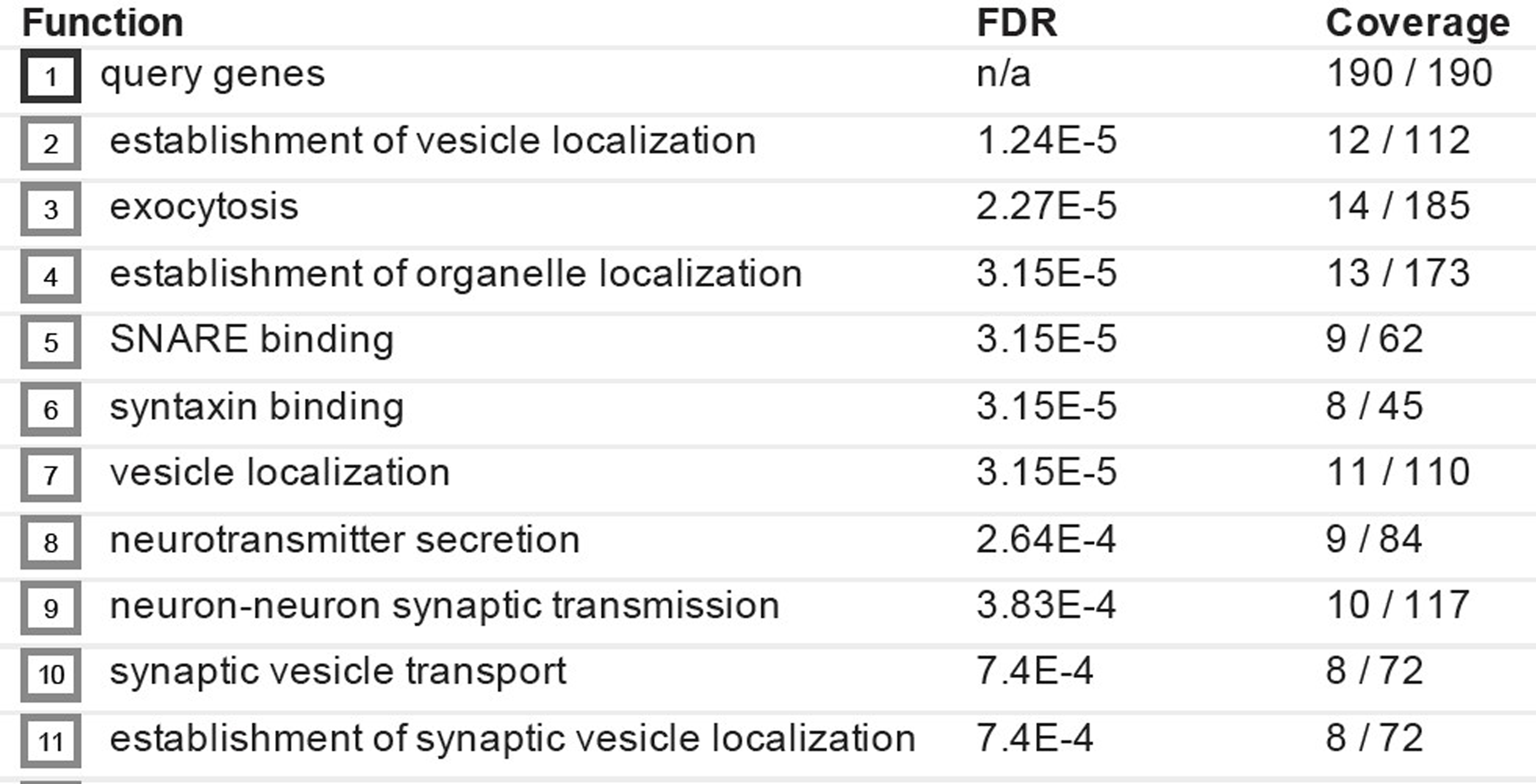

Supplement: Supplementary Table 4 [file tp201666x12.tif]

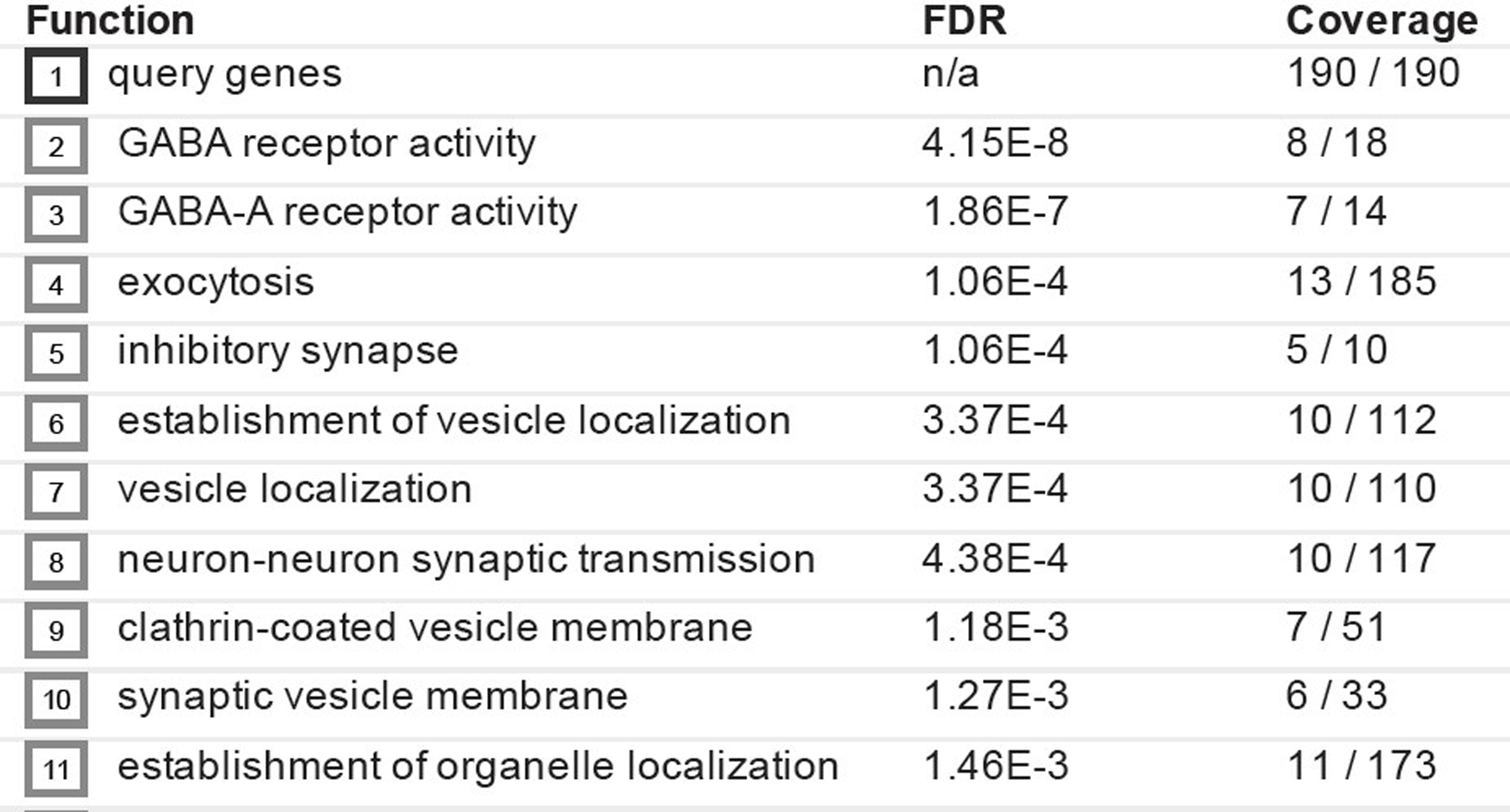

Supplement: Supplementary Table 5 [file tp201666x13.tif]
